# Treatment biomarkers for ADHD: Taking stock and moving forward

**DOI:** 10.1038/s41398-022-02207-2

**Published:** 2022-10-12

**Authors:** Giorgia Michelini, Luke J. Norman, Philip Shaw, Sandra K. Loo

**Affiliations:** 1 Department of Biological and Experimental Psychology, School of Biological and Behavioural Sciences, Queen Mary University of London, London, UK; 2 Department of Psychiatry and Biobehavioral Sciences, Semel Institute for Neuroscience and Human Behavior, University of California Los Angeles, Los Angeles, CA USA; 3 Office of the Clinical Director, NIMH, Bethesda, MD USA; 4 Section on Neurobehavioral and Clinical Research, Social and Behavioral Research Branch, National Human Genome Research Institute, NIH, Bethesda, MD USA

**Keywords:** Biomarkers, Neuroscience

## Abstract

The development of treatment biomarkers for psychiatric disorders has been challenging, particularly for heterogeneous neurodevelopmental conditions such as attention-deficit/hyperactivity disorder (ADHD). Promising findings are also rarely translated into clinical practice, especially with regard to treatment decisions and development of novel treatments. Despite this slow progress, the available neuroimaging, electrophysiological (EEG) and genetic literature provides a solid foundation for biomarker discovery. This article gives an updated review of promising treatment biomarkers for ADHD which may enhance personalized medicine and novel treatment development. The available literature points to promising pre-treatment profiles predicting efficacy of various pharmacological and non-pharmacological treatments for ADHD. These candidate predictive biomarkers, particularly those based on low-cost and non-invasive EEG assessments, show promise for the future stratification of patients to specific treatments. Studies with repeated biomarker assessments further show that different treatments produce distinct changes in brain profiles, which track treatment-related clinical improvements. These candidate monitoring/response biomarkers may aid future monitoring of treatment effects and point to mechanistic targets for novel treatments, such as neurotherapies. Nevertheless, existing research does not support any immediate clinical applications of treatment biomarkers for ADHD. Key barriers are the paucity of replications and external validations, the use of small and homogeneous samples of predominantly White children, and practical limitations, including the cost and technical requirements of biomarker assessments and their unknown feasibility and acceptability for people with ADHD. We conclude with a discussion of future directions and methodological changes to promote clinical translation and enhance personalized treatment decisions for diverse groups of individuals with ADHD.

## Introduction

Biomarker discovery for psychiatric disorders and symptoms has been challenging, despite a clear need to guide clinical decisions [1–3] and a vast literature examining neurobiological underpinnings of diagnoses, dimensional constructs (i.e., Research Domain Criteria [RDoC]) [4, 5], developmental trajectories [6, 7], and treatment response [8, 9]. Furthermore, promising findings are rarely translated to clinical practice, in academic medical hospitals or clinics or even further, to community clinical settings. This has been true for nearly all psychiatric disorders, yet particularly true for neurodevelopmental disorders such as attention-deficit/hyperactivity disorder (ADHD), which is likely to have multiple etiological and neurobiological pathways and whose benchmarks for “typical” and “pathological” are moving targets due to population-level variability in behavioral, cognitive, and brain maturation rates [3, 10]. The lack of biomarker translation is especially evident in the slow development of novel treatments for ADHD (and many other disorders) [11], where the gold-standard of treatment, psychostimulant medications, has been the same for over 50 years. Despite the slow progress, the considerable scientific efforts and substantial literature base provide a promising foundation for biomarker discovery. The goal of this article is to give an updated narrative review of promising biomarkers for ADHD treatment response along with methodological changes that may assist with clinical translation to enhance personalized medicine and novel treatment development.

**Fig. 1:**
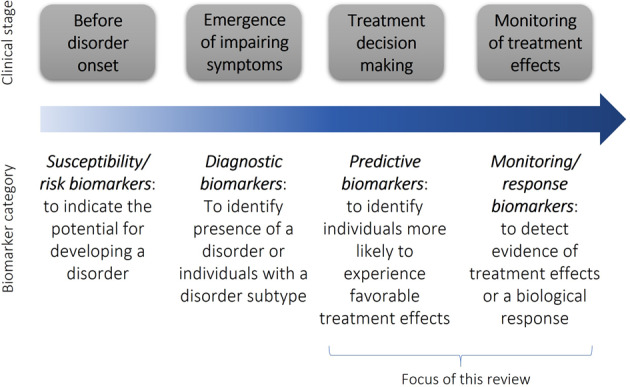
Timeline showing points through clinical course where different categories of biomarkers (adapted from the Food and Drug Administration Biomarkers, EndpointS, and other Tools Resource [13]) have the potential to impact clinical practices in psychiatry. This review focuses specifically on treatment biomarkers.

We first briefly discuss biomarker definitions and categories to provide a framework for the review. In 2001, the NIH Biomarkers Definitions Working Group defined a biomarker as “a characteristic that is objectively measured and evaluated as an indicator of normal biologic processes, pathologic processes, or biological responses to a therapeutic intervention. A biomarker can be a physiologic, pathologic, or anatomic characteristic or measurement that is thought to relate to some aspect of normal or abnormal biologic function or process” [12]. While there have been several variants on this basic theme, particularly as applied to various biomarkers of types (e.g., blood biomarkers) and disease states (e.g., cancer biomarkers or psychiatric biomarkers), a recent outline of biomarker categories by the Food and Drug Administration (FDA) Biomarkers, EndpointS and other Tools (BEST) [13] Resource will be used as a framework for the current review (Fig. 1). Literature on diagnostic and susceptibility/risk biomarkers are outside the scope of this paper (recent reviews can be found elsewhere [3, 14–16]). We thus focus on categories relevant to treatment decision making (i.e., predictive and monitoring/ response biomarkers). We highlight key findings by providing examples from strong, methodologically rigorous studies, particularly on pharmacological treatments, along with methodological barriers to clinical translation.

## Predictive biomarkers

Given the wide variability in effectiveness and tolerability of available treatments, an important motivation for developing biomarkers has been to identify measures parsing this variability and aiding personalized treatment decisions. This is especially needed for treatments such as non-stimulants and non-pharmacological options, where effects may not be observed until weeks after treatment initiation. Here, we use the term “predictive biomarkers” based on BEST guidelines [13] (Fig. 1), but recognize that studies have also referred to these measures as “prognostic biomarkers” [17].

### Structural and functional neuroimaging

Pre-treatment subcortical volumes have been associated with treatment response to methylphenidate (MPH), with responders showing smaller volumes than non-responders [18] in one study but greater gray matter concentration [19] in another study (Table 1). A single study using diffusion-weighted imaging found that a machine learning algorithm could predict better MPH response from higher values of local efficiency (reflecting how efficiently information can be distributed between a brain region and its neighbors) within the thalamus, precentral gyrus and superior frontal gyrus [20]. Although the directions of the patterns are mixed, measures implicated in the pathophysiology of ADHD have been found to have potential predictive value with treatment effects.

**Table 1:** Details of MRI studies investigating candidate predictive/prognostic biomarkers for treatment response.

Authors, year	Country	N ADHD	N Controls	Age	% Male	% White	Design	Candidate biomarker(s)	Key findings
Griffiths et al. [20]	Australia	37 (19 had taken psychostimulants in the previous 6-months, and underwent a washout period)	26	*M* = 13.29 SD = 2.64	70%	Not reported	6-week open label study of MPH, pre-treatment neuroimaging	White matter local efficiency (graph theoretical measure of how efficiently information can be distributed between a brain region and its neighbors)	Support vector machine learning algorithm applied to multivariate measures of local efficiency predict treatment response assessed at 6-weeks using the ADHD-RS-IV. The most predictive features were higher local efficiency of the thalamus, precentral gyrus and DLPFC.
Hong et al. [24]	South Korea	83 (medication-free for >4 weeks, and with no history of long-term treatment for ADHD, defined as medicated >6 months)	22	*M* = 9.63 SD = 2.61	25%	Not reported	8-week RCT of MPH, pre-treatment neuroimaging	Resting-state connectivity assessed using striatal seeds	Treatment responders (*n* = 48), defined according to the CGI-I at 8-weeks, showed greater pre-treatment connectivity between striatal seeds and orbitofrontal, cingulo-opercular and middle and medial temporal regions than did non-responders.
Kim et al. [18]	South Korea	67 (medication-free for >4 weeks, and with no history of long-term treatment for ADHD, defined as medicated >6 months)	25	*M* = 9.83 SD = 2.5	25%	Not reported	8-week RCT of MPH, pre-treatment neuroimaging	Subcortical volume	Responders had smaller volumes in bilateral amygdala and hippocampal subregions and right thalamus than non-responders.
Lam et al. [29]	UK	31 (24 subjects were receiving stable medication)	0	*M* = 13.90 SD = 1.58	100%	Not reported	2-week real-time fMRI neurofeedback of the right IFG vs. neurofeedback of a control para-hippocampal region. Single-blind RCT	Brain activation during inhibitory control task as a predictor of neurofeedback learning	Better neurofeedback learning was associated with pre-treatment activation in left IFG/insula and striatum during the fMRI stop task.
Mizuno et al. [83]	Japan	27 (all medication-free for >5 times half-lives)	49	*M* = 10.96, SD = 2.14	100%	Not reported	Double-blind, placebo-controlled, crossover design comparing single-dose MPH and placebo	Dynamic resting-state functional connectivity	Dynamic network interactions under placebo predicted individual differences in sustained attention improvements under MPH.
Moreno et al. [19]	Spain	27 (all treatment naïve)	0	*M* = 9.33 SD = 2.49	70%	Not reported	4-week open-label trial of MPH, pre-treatment neuroimaging	Subcortical gray matter concentration	Treatment responders, as defined via clinical interview and administration of CGI and CGAS, showed greater gray matter concentration within the nucleus accumbens and caudate compared with non-responders.
Norman et al. [22]	USA	110 (medicated)	142	age range, 6–17 years	65%	Not reported	Naturalistic longitudinal study of chronically medicated subjects, including up to 5 assessments. Scanning was performed during washout period.	Resting-state connectivity within and between cingulo-opercular, default mode and subcortical networks was assessed while subjects were off medication.	ADHD symptoms were rated on and off medication using the DICA-IV interview for parents. Non-responders showed developmentally atypical increases in cingulo-opercular connectivity with age, while responders showed a developmental trajectory that tracked that of the controls.
Peterson et al. [21]	USA	16 (psychostimulant responders)	20	*M* = 13.71 SD = 2.85	69%	94%	Non-blinded study of chronically medicated psychostimulant responders with ADHD. Subjects were scanned on and off medication	Brain activation was assessed during a stop task both on and off medication	Off-medication left lateral prefrontal cortex activation correlated with differences in total ADHD symptoms, which were assessed using on and off medication versions of the CPRS in youth with ADHD.
Schulz et al. [27]	USA	36 (medication-naïve)	0	*M* = 11.0 SD = 2.4	83%	Not reported	8-week MPH/ATX randomized cross-over design, pre-treatment	Brain activation during a go/no-go task	Greater pre-treatment caudate activation was associated with a better treatment response to MPH, but a worse response to ATX, as assessed using ADHD-RS-IV.

*ADHD* attention deficit hyperactivity disorder, *ADHD-RS-IV* ADHD Rating Scale-IV, *ATX* atomoxetine, *CGAS* Children’s Global Assessment Scale, *CGI* Clinical Global Impressions scale, *CPRS* Conners’ Parent Rating Scale, *DICA-IV* Diagnostic Interview for Children and Adolescents – IV; *fMRI* functional magnetic resonance imaging; *IFG* inferior prefrontal gyrus, *MPH* methylphenidate, *MRI* magnetic resonance imaging, *RCT* randomized controlled trial.

With regard to functional MRI, studies have shown that greater left lateral prefrontal cortex activation during a Stroop task [21] and age-related increases in within-network cingulo-opercular connectivity tracking the developmental trajectory of neurotypical controls [22] were associated with improvement in ADHD symptoms as assessed while subjects were on versus off medication. One of these studies in particular suggests that predictive biomarkers of treatment response may be developmentally sensitive, requiring repeated assessments of developmental change [22]. Importantly, both studies used naturalistic, observational designs of chronically medicated youth scanned off medication, and may not generalize to very poor treatment responders, who are more likely to cease treatment [21, 22]. Moreover, since long-term medication exposure may produce changes in brain patterns, findings may not apply to individuals assessed prior to treatment [23]. These issues can be overcome by clinical trials. In one such study, lower pre-treatment connectivity from striatal regions to orbitofrontal, cingulo-opercular and middle and medial temporal regions was associated with better treatment outcomes after 8-week MPH treatment [24]. Studies have further tested whether acute changes (particularly with short-acting psychostimulants) may predict symptom improvement over longer timeframes [22]. To our knowledge, only one fMRI study has adopted such a design in a small sample (*N* = 7), reporting that greater decreases in regional homogeneity (i.e., local coherence in fluctuating BOLD signals, or local connectivity) within right postcentral gyrus and superior parietal lobe following a single MPH dose predicted lower ADHD severity at 8 weeks [25].

While most studies focused on a single form of treatment, neuroimaging profiles predicting differential response to different treatments may be more useful to guide treatment decisions at the individual level (i.e., treatment stratification [26]). A notable study of this type, using a double-blind, cross-over randomized controlled design comparing 8-week MPH vs. atomoxetine (ATX) treatment, found that greater pre-treatment caudate activation predicted better treatment response to MPH, but worse response to ATX [27]. These findings suggest that the identified fMRI patterns, if replicated, may be valuable for predicting response to different treatments.

In addition to potential applications in treatment allocation algorithms, an aim of biomarker research is to develop novel neurotherapies targeting neural processes associated with a disorder or modulated by existing treatments [11]. The most elegant neuroimaging example is provided by studies investigating fMRI neurofeedback of the right inferior frontal gyrus (IFG) as a novel ADHD treatment [28–30], guided by meta-analytic evidence that the right IFG is under-activated in individuals with ADHD [31, 32], but upregulated by ADHD medications [9, 31, 32]. Although clinical improvements over 2 weeks were observed both with rIFG-neurofeedback and a control neurofeedback condition (para-hippocampal region), only active rIFG-neurofeedback showed evidence of improvements in everyday life (i.e., learning retention) after 11 months [28]. Individual differences in neurofeedback learning were associated with greater pre-treatment inferior frontal and striatal activation during an inhibitory control task [29].

Overall, while the ability of single pre-treatment brain scans to guide clinical decision making is an important goal, the current neuroimaging literature does not point to any immediate clinical applications of neuroimaging biomarkers due to inconsistent findings and small sample sizes (Table 1). Further, the high costs, contraindications and low tolerance of participants’ movement of MRI are considerable barriers to the future implementation of neuroimaging biomarkers in clinical settings (particularly for highly hyperactive children), and will require substantial methodological innovation (e.g., shorter scans, better motion corrections). Finally, the methodology for testing promising findings as predictive biomarkers must evolve to be clinically useful. For example, the right IFG-neurofeedback findings [28–30] should be quantified for a threshold value (of activation) that maximally identifies treatment response, and then that value should be used as an inclusion criterion or to stratify participants in subsequent independent prospective trials to determine its predictive validity. This last step has not been implemented in any neuroimaging studies of predictive biomarkers to date but is necessary to develop a biomarker that can be used by others in clinical settings.

### EEG

EEG has been one of the main techniques to investigate the neural mechanisms of ADHD treatment response [8, 33]. Compared to fMRI, EEG recordings allow the direct investigation of brain activity with greater temporal resolution, but less accurate spatial precision [1, 17]. EEG is also less expensive and more tolerant of participants’ movement, making it easier to collect large clinical samples over time for the development of treatment biomarkers [34].

The most consistent findings suggest that higher spectral power in the theta band (4–7 Herz [Hz]) during resting states (eyes open or closed) is associated with better clinical outcomes following stimulant treatment [17, 35, 36] (Table 2). A common interpretation of this finding is that lower pre-treatment levels of arousal and vigilance, commonly displayed by children with ADHD [37], predict better response. In some studies, excess theta power in responders was accompanied by lower power in faster frequencies, especially beta (generally 13–25 Hz), and elevated theta-to-beta-ratio (TBR) [35, 36]. Yet, an association between TBR and treatment outcome was not replicated in a larger, more recent study [38], suggesting that TBR may have questionable predictive utility, besides its well-documented limited diagnostic properties [39, 40]. Further, event-related potential (ERP) studies found that more intact auditory P3 [41], cue P3 and contingent negative variation [36, 42] amplitudes, reduced no-go P3 amplitudes [36, 42], and greater change in P3 amplitude after a single stimulant dose predicted better stimulant response [42, 43]. Two notable studies also tested the combined predictive effects of spectral power and ERPs on treatment response [36, 42], accounting for shared variance between various measures. In a multivariate model, higher cue P3, smaller no-go P3, and excess theta power were the only significant predictors of stimulant response [36]. An aggregate index combining EEG/ERP and performance measures predicted treatment response with 88% specificity and 86% sensitivity [42].

**Table 2:** Details of EEG studies investigating candidate predictive/prognostic biomarkers for treatment response.

Authors, year	Country	N ADHD	N Control	Age	% Male	% White	Design	Candidate biomarker(s)	Key findings
Arns et al. [38]	USA, Australia, Netherlands	336 (all medication free for >7 days)	158	*M* = 12	72%	Not reported	6-week MPH, open label	iAPF during rest	Lower pre-treatment frontal iAPF in male adolescent non-responders relative to responders. No difference in pre-treatment TBR, age, medication dosage, ADHD severity.
Chiarenza et al. [44]	Italy	61 (all medication free for >5 times half-lives)	Not reported (reference database)	*M* = 10.4, SD = 2.9	85%	Not reported	12-month ATX, open label	Absolute power across frequency bands during rest	Higher pre-treatment frontal alpha and fronto-temporal delta and theta power in responders relative to controls. Higher pre-treatment absolute power in all frequency bands (especially frontal and central) in non-responders relative to controls.
Griffiths et al. [45]	Australia	52 (all medication free)	52	*M* = 11.9, SD = 2.5	83%	Not reported	6-week ATX vs. placebo, cross-over RCT	N2 amplitude during an auditory oddball task	Lower pre-treatment N2 amplitudes (especially right fronto-central) in responders relative to non-responders and controls. N2 predicted responders vs. non-responders with specificity = 80.8% and sensitivity = 47.1% in a leave-one-out cross validation analyses.
Krepel et al.[50]	Netherlands, Germany, Australia	136 (43 medication free, 93 medicated)	0	*M* = 24.9, SD = 14.9	89%	Not reported	QEEG-informed NF (NF protocols based on individual EEG), open label	P3 amplitude (females), iAPF (males) during rest	Shorter pre-treatment P3 latencies in girls/women who remitted; lower pre-treatment iAPFs in boys/men who remitted.
Leuchter et al. [47]	USA	44 (medication free for >10 days)	0	Range = 18–30	Not reported	Not reported	12-week ATX vs. placebo RCT	Change in theta cordance during rest at 1-week post-treatment	Lower left temporoparietal theta cordance at 1-week post-treatment in ATX responders relative to non-responders. No difference between placebo responders and non-responders. Lower theta cordance predicted improvement in ADHD symptoms and quality of life. No association between absolute and relative power measures and clinical outcomes.
Loo et al. [49]	USA	51 (all medication free for >1 month)	0	*M* = 10.3, SD = 1.4	67%	Not reported	4-week active vs. sham TNS, RCT	Theta and alpha power during rest	Lower pre-treatment right-frontal theta and alpha power in responders relative to non-responders. Treatment-related change right-frontal theta predicted response AUC = 00.81.
Luo et al. [56]	China	121	0	*M* = 8.9, range= 7.1–12.3	83%	Not reported	3-month remote computerized cognitive, NF, and combined training, RCT	Relative alpha power during rest	Pre-training relative alpha power correlated positively with ADHD improvements.
Michelini et al. [57]	USA	207 (all medication naïve or medication free for >5 times half-lives)	0	*M* = 10.1, SD = 2.1	68%	83%	8-week MPH, GUAN, MPH + GUAN, RCT	Event-related midfrontal beta power localized in the ACC during the Sternberg spatial working memory task with encoding, maintenance and retrieval phases	Weaker mid-frontal beta power modulations across task phases predicted greater ADHD improvements with MPH + GUAN. Stronger mid-frontal beta power modulations predicted clinical improvements with GUAN (during retrieval) and binary response with MPH (during encoding). Mid-frontal beta & clinical measures at pre-treatment explained *R*^2^ = 0.41 in MPH + GUAN and *R*^2^ = 0.34 in GUAN groups; clinical measures alone explained *R*^2^ = 0.21 in MPH + GUAN and *R*^2^ = 0.14 in GUAN groups.
Ogrim et al. [36]	Norway	98	90	Range = 7–17	67%	Not reported	4-week MPH or DEX, open label	Theta power, contingent negative variation, cue P3 and no-go-P3 during a cued go/no-go task	Higher pre-treatment frontal theta power and cue P3 amplitudes, more negative contingent negative variation amplitude and lower posterior alpha power and no-go P3 amplitudes in responders relative to no-responders. Cue P3, no-go P3, and excess theta predicted response in a multivariate model. No difference in iAPF between responders and non-responders.
Ogrim et al. [42]	Norway	87	0	Range = 7–17	69%	Not reported	Single dose and 4-week MPH or DEX, open label	Theta/alpha power, no-go P3, contingent negative variation during cued go/no-go task	Higher pre-treatment Cz theta/alpha ratio, lower pre-treatment no-go P3, higher single-dose change in no-go P3 and lower single-dose change in contingent negative variation in responders than non-responders. An aggregate index of ERP and behavioral predictors yielded AUC = 91%, sensitivity=86%, specificity = 88%.
Sangal & Sangal [46]	Not reported (probably USA)	17 (all medication naïve or medication free for >5 times half-lives)	0	M = 10.9, SD = 3.0	71%	82%	10-week ATX, open label	Auditory P3 amplitude during visual and oddball tasks	Higher pre-treatment P3 amplitude across regions in responders relative to non-responders, yielding PPV = 0.88 and NPV = 0.67.
Sangal & Sangal [41]	Not reported (probably USA)	58 (all medication free for >1 month)	0	M = 10.5, SD = 2.1	72%	Not reported	4-week ATX vs. MPH, cross-over RCT	Auditory P3 amplitude during visual and oddball tasks	Greater pre-treatment P3 amplitude across regions in ATX responders relative to non-responders; greater pre-treatment P3 amplitude at right temporal region in MPH responders relative to non-responders.
Sari Gokten et al. [35]	Turkey	51	0	M = 8.57, SD = 1.75	82%	Not reported	13-month MPH, open label	Delta, theta, gamma power, delta/beta, and TBR during rest	Higher pre-treatment delta power at F8, theta power at Fz, F4, C3, Cz, T5, gamma power at T6, lower beta power at F8 and P3, delta/beta ratio at F8 and TBR at F8, F3, Fz, F4, C3, Cz, P3, and T5 predicted greater hyperactivity improvement. Theta power at Cz and T5 and TBR at C3, Cz, and T5 also accurately classified responders vs. non-responders in logistic regressions.
Singh et al. [48]	India	50 (all medication naïve)	0	Range = 6–14	80%	Not reported	6-week ATX, open label	Change in theta cordance during rest at 1-week post-treatment	Greater decrease in left temporoparietal theta cordance at 1 week in responders relative to on-responders. No difference between pre-treatment and 1 week in non-responders.
Voetterl et al. [52]	Netherlands, Australia, USA	Transfer: 336 MPH & 136 NF; validation: 41 MPH & 71 NF. Exploration: 55 GUAN & 47 ATX	0	Range = 7–15	100%	Not reported	Various duration, MPH, multimodal NF with with sleep coaching, GUAN, ATX, open label or RCTs	iAPF during rest	Transfer phase: predicted gain in normalized remission of 17% to 30% after stratifying boys with a higher iAPF to MPH and boys with a lower iAPF to multimodal NF, respectively. Blinded out-of-sample validations: predicted gain in stratified normalized remission of 36% and 29%, respectively. Exploration phase: higher iAPF predicted remission with GUAN and lower iAPF predicted remission with ATX.
Young et al. [43]	Not reported (probably Australia)	35	0	M = 13.3, SD = 2.48	58%	Not reported	Single dose and 6-month MPH, open label	P3b during auditory oddball task	Acute P3b amplitude changes accurately predicted treatment outcome in 81% of cases.

*ADHD* attention deficit hyperactivity disorder, *ATX* atomoxetine, *AUC* area under the curve statistic, *EEG* electroencephalography, *DEX* dexamphetamine, *ERP* event relate potential, *GUAN* guanfacine, *MPH* methylphenidate, *iAPF* individual alpha peak frequency, *M* mean age, *NF* neurofeedback, *NPV* negative predictive value, *PPV* positive predictive value, *SD* standard deviation, *TNS* trigeminal nerve stimulation.

Fewer studies focused on non-stimulant medications. A 12-month study of ATX found broad pre-treatment increases in power in children classified as non-responders relative to controls, whereas responders only showed elevations in slower frequencies such as the delta (<4 Hz), theta and alpha (8–12 Hz) bands [44]. Lower pre-treatment N2 amplitudes [45] and greater pre-treatment P3 amplitudes [41, 46] during auditory tasks have been associated with better response after 6–10 weeks of ATX treatment, although non-significant P3 effects were also reported [45]. Both in children and in adults, greater change in temporo-parietal theta cordance (a measure of regional spectral power) at 1 week predicted clinical outcomes at 6–12 weeks [47, 48].

Although most available studies of EEG predictive biomarkers have investigated pharmacological treatments, a notable non-pharmacological example is a novel 4-week double-blind, sham-controlled trigeminal nerve stimulation (TNS) trial, where lower pre-treatment resting-state right-frontal theta and alpha power predicted greater clinical improvement [49]. Furthermore, EEG power, coupled with deficits on a behavioral rating of executive functioning, had an area under the curve (AUC) of 0.81, suggesting good prediction of TNS treatment response. Since these findings come from the first RCT testing TNS for ADHD, replication in future larger trials is needed. Another non-pharmacological study [50] found that, compared to non-remitters, girls and women whose ADHD symptoms remitted following quantitative EEG-informed neurofeedback showed shorter frontal P3 latencies during an auditory task, whereas boys and men who remitted had lower individual alpha peak frequency (iAPF; i.e., the frequency at which an individual’s alpha activity oscillates, with slower profiles potentially reflecting reduced thalamo-cortical information flow [51, 52]). Given the relatively inconclusive evidence on neurofeedback efficacy for ADHD at the group level [53–55], these studies may help to identify biologically-distinct ADHD subgroups who respond better to this treatment.

Only a few studies tested EEG predictors of more than one type of treatment [52, 56, 57]. An 8-week randomized controlled trial (RCT) comparing MPH, guanfacine and their combination found that event-related EEG power profiles during a working memory task showed treatment-specific associations with clinical improvements [57]. Specifically, better treatment outcome was predicted by weaker mid-frontal beta power modulations localized in the anterior cingulate cortex (ACC) in children treated with combined MPH + guanfacine, but by stronger modulations in children treated with MPH or guanfacine. Together, EEG measures explained twice as much variance in treatment outcome than clinical measures alone in children treated with guanfacine and combined treatments. These findings, while awaiting replication in independent samples, suggest that EEG profiles could supplement clinical information to aid future personalized treatment decisions.

Another noteworthy exemplar based on different treatments is a recent multi-sample study of iAPF [52]. Guided by previous literature showing associations of slow iAPF with worse stimulant response [38, 58] but better neurofeedback response [50], particularly in boys and men, this study tested whether iAPF differentially predicted ADHD remission following MPH and multimodal neurofeedback treatments in boys with ADHD [52]. After developing and validating iAPF as a neurobiologically plausible biomarker in a large transdiagnostic sample, this study found that iAPF was able to stratify children with ADHD to MPH (high iAPF) or multimodal neurofeedback (low iAPF) with gains in remission of 17–30% relative to observed remission rates. These results were corroborated in blinded, out-of-sample validations, with predictive gains of 29–36%. Additional exploratory analyses showed that iAPF predicted remission to guanfacine (high iAPF) and ATX (low iAPF). These findings point to iAPF as a robust biomarker able to predict treatment outcome across independent samples. As stated above for neuroimaging biomarkers, an important next step to inform future personalized treatment approaches would be to carry out a prospective study using iAPF to stratify individuals with ADHD to different treatments.

To summarize, while most of the EEG profiles predicting treatment outcomes have been examined only for stimulants, right frontal resting state spectral power, event-related power related to working memory and iAPF emerge as candidate predictive biomarkers that may aid future stratification and prediction of response to different treatments. Future studies will need to apply out-of-sample stratification and validation approaches prospectively to a wider portion of the ADHD population, treatments and EEG profiles.

### Genetics

Genetic studies have also investigated biomarkers predicting treatment response, particularly to MPH (Table 3). The first wave of studies took a candidate gene approach focused on single nucleotides polymorphisms (SNPs) and variable number tandem repeat (VNTR) within or proximal to monoaminergic genes [59, 60], as ADHD medications act on dopaminergic, serotonergic and adrenergic systems. A meta-analysis found that many candidate variants predicted MPH response with odds ratios (ORs) around 1.5–3 [60], including a VNTR within the SLC6A3 gene encoding the dopamine transporter, SNPs tagging the gene coding for the norepinephrine transporter (SLC6A2), variants within the DRD4 gene altering receptor expression and near the ADRA2 gene coding for the alpha-2-adrenergic receptor, and a SNP within the enzyme Catechol-o-methyltransferase (COMT) involved in degrading catecholamines.

**Table 3:** Details of genetic studies investigating candidate predictive biomarkers for treatment response.

Authors, year	Country	N ADHD	N Control	Age	% Male	% White	Design	Candidate biomarker(s)	Key findings
Brikell et al. [64]	Denmark	Starting MPH (*N* = 7427) stopping MPH (*N* = 3370) or switching to non-stimulant (*N* = 1137) over 2 years	0	Range = 3–32 (age at first diagnosis)	71%	100%	Linked genetic and medical records	16q23.3 locus	16q23.3 locus (containing genes and variants associated with a range of neuropsychiatric phenotypes such as seasonal depression, alcohol intake and cerebellar volume) was significantly associated with switching medication. No genome-wide significant associations for starting or stopping treatment. No associations of ADHD PRS with treatment outcomes; bipolar disorder PRS and schizophrenia PRS predicted stopping stimulant medication.
Elia et al. [65]	Europe, USA	1013 (discovery sample) + 2493 (replication sample)	4105 + 9222(replication sample)	Range = 6–18	Not reported	100%	Multiple samples of ADHD cases and controls	CNVs within metabotropic glutamate receptor network genes	ADHD-associated CNVs were concentrated in genes within a network of metabotropic glutamate receptor genes, affecting 11.3% of ADHD cases compared to 1.2% of unaffected controls.
Elia et al. [66]	USA	30 (all harboring mutations in metabotropic glutamate receptor network genes)	0	Range = 12–17	66%	50%	5-week, open-label, single-blind, placebo-controlled trial of the metabotropic glutamate activator receptor fasoracetam	CNVs within metabotropic glutamate receptor network genes	Individuals who harbored CNVs within this glutamatergic gene network had better therapeutic response.
Gul et al. [59]	Turkey	100	80	Range = 6–15	66%	Not reported	2-month ATX, open label	A SNP (rs3785143) tagging the SLC6A2 gene	rs3785143 showed association with ATX response, with CC homozygotes showing superior response. OR ~3 with wide confidence intervals (1.1–13.4)
Myer et al. [60]	Multiple	3647	0	*M* = 9.5, range=4–13	83%	Multiple	Meta-analysis of candidate genes studies predicting MPH response	10 repeat VNTR within the SLC6A3 gene; SNPs tagging the SLC6A2 gene	Homozygotes for the 10 repeat VNTR within the SLC6A3 gene encoding the dopamine transporter targeted by MOH show worse response. SNPs tagging the gene coding for the norepinephrine transposer (SLC6A2) were tied to altered responsivity. Response was also moderated by variants within the DRD4 gene, that in silico alters receptor expression, and near the ADRA2 gene, coding for the alpha-2-adrenergic receptor. A polymorphism within the enzyme, COMT, which reduces its potency in degrading catecholamines, was tied to increased response.
Pagerols et al. [62]	Spain (children), Brazil (adult)	173 children (discovery sample) + 189 adults (replication sample)	0	Mean=9.6, SD = 2.9	84% (children), not reported (adults)	100%	PRS for MPH response derived in children and tested in adults to predict MPH response at 8 weeks	None	No genome-wise significant hits in children and no association between the PRS for treatment response in adults. The set of genes containing SNPs nominally associated with response (*p* < 0.05) was significantly enriched for candidates previously studied in ADHD or treatment outcome.
Zhong et al. [63]	China	241 (most medication naïve, 13 medication free for >1 week)	0	Mean=9.2, SD = 2.2	85%	Not reported	8/12-week MPH or ATX, open label	ADHD PRS	There were no genome-wide significant hits for treatment response. PRS for ADHD was found to predict a favorable response, explaining 2% of the variance.

*ADHD* attention deficit hyperactivity disorder, *ATX* atomoxetine, *CNV* copy number variant; *COMT* Catechol-o-methyltransferase, *GWAS* genome-wide association study, *M* mean age, *MPH* methylphenidate; *PRS* polygenic risk score; *RCT* randomized controlled trial, *SD* standard deviation, *SNP* single nucleotides polymorphism, *VNTR* variable number tandem repeat.

Given the known limitations of candidate gene approaches [61], more recent work has focused on genome-wide association studies (GWAS). As large sample sizes are needed in GWAS of complex phenotypes such as medication response, studies including less than 250 participants found no genome-wide significant SNPs tied to medication response [62, 63]. More promising results come from a large Danish study that linked genotype data with medical records, allowing a well-powered GWAS of starting (*N* = 7427) or stopping (*N* = 3370) MPH treatment or switching to non-stimulants (*N* = 1137) over 2 years [64]. While no genome-wide significant associations emerged for starting or stopping treatment, a locus on 16q23.3 containing genes associated with many neuropsychiatric phenotypes was associated with switching medications, likely due to poor efficacy. In addition to common genetic variants, a few studies have examined rarer genetic variants, such as copy number variants (CNVs; these represent large scale genomic duplications or deletions). One study reported that ADHD-associated CNVs were concentrated in genes within a network of metabotropic glutamate receptor genes, affecting 11.3% of ADHD cases compared to 1.2% of neurotypical controls [65]. In a subsequent open-label trial, individuals who harbored CNVs within this glutamatergic gene network had better therapeutic response to an activator of this glutamate receptor, fasoracetam [66].

Since each individual genetic marker only explains a tiny fraction of treatment response, three studies have investigated aggregate measures of genetic risk using polygenic risk scores (PRS) (i.e., the sum of alleles across the genome weighted by their effect size). PRS for ADHD was found to predict a favorable response to ADHD medication (MPH or ATX), explaining around 2% of the variance [63]. The aforementioned Danish study did not find the PRS for ADHD to be associated with treatment outcomes [64], consistent with recent evidence that genes implicated in the pathogenesis of ADHD do not overlap with genes encoding targets of stimulants and ATX [67]. However, PRS for bipolar disorder and schizophrenia increased the likelihood of stopping stimulant medication (~5–7% of the variance) [64], mirroring findings that comorbid bipolar or psychotic disorders are associated with poor treatment response and adverse events. Finally, a PRS for MPH response derived in a childhood cohort did not significantly predict response in an independent adult cohort [62], with several interesting nominally significant signals that may be worthy of future exploration.

Additionally, there has been interest in genetic markers that lie within the cytochrome P450 superfamily of enzymes. The cytochrome P450 system largely determines the metabolism of ATX and varies widely between individuals by genotype, and by race and ethnicity [68]. Pharmacokinetic genetic studies have particularly focused on the cytochrome P450,2D6 (CYP2D6), contrasting individuals with different genotypes leading to ultrarapid, extensive and poor metabolism of ATX. Poor metabolizers show up to 9 times less plasma clearance of ATX than the extensive metabolizers, creating greater exposure to the drug. In turn this raises the question of whether dosage adjustment based on CYP2D6 genotype may help avoid adverse side effects, at least for those taking high doses of ATX.

Together, initial findings suggest that stratifying individuals based on genetic markers, such as PRS and CNVs, may be useful to guide future treatment choice in a mechanistic manner. Future studies will need to use much larger samples, consider possible multiplicative effects of different genetic markers, investigate the effects of genetic biomarkers on response to multiple pharmacological and non-pharmacological treatments, and test the predictive utility of genetic biomarkers in combination with other biomarkers, such as the promising neuroimaging and EEG biomarkers discussed above.

## Monitoring and response/pharmacodynamic biomarkers

Another major application of treatment biomarkers is for treatment monitoring, with repeated biomarker assessments performed before and after treatment, in response to acute doses or over longer periods. Neuroimaging and EEG biomarkers reviewed in this section are consistent with the BEST monitoring biomarker category and response/pharmacodynamic biomarker category [13] (also sometimes called “pharmacokinetic biomarkers”) (Fig. 1).

### Structural and functional neuroimaging

Both acute and longer-term treatment effects on brain patterns have been documented using structural and functional magnetic resonance imaging in ADHD samples (Table 4). Changes in structural neuroimaging markers following treatment with MPH have been studied as part of a 16-week double-blind, randomized, placebo-controlled trial of boys and young men with ADHD [69, 70]. One report from this trial showed wide-spread time-by-medication-by-age interaction effects in left hemisphere white matter, which were driven by increases in fractional anisotropy among medicated children [69]. A report from the same trial described increasing cortical thickness in medicated children within right medial frontal cortex, which contrasted with the cortical thinning that was observed in the placebo group. No significant results were found for the adult groups, and structural brain changes were not associated with clinical improvement [70]. Similarly, a double-blind randomized placebo-controlled trial of *N* = 131 adults with ADHD who underwent 12 months of MPH treatment reported no significant treatment-related changes in gray matter volume [71].

**Table 4:** Details of neuroimaging studies of candidate monitoring/response biomarkers.

Authors, year	Country	N ADHD	N Controls	Age	% Male	% White	Design	Candidate biomarker(s)	Key findings
Alegria et al. [28]	UK	31 (24 receiving stable medication)	0	*M* = 13.90 SD = 1.58	100%	Not reported	2-week real-time fMRI neurofeedback of the right IFG vs. a control para-hippocampal region. Single-blind RCT.	Neurofeedback transfer effect (IFG upregulation in the absence of feedback) and brain activation during inhibitory control task	Improvements in ADHD symptoms over 2 weeks were observed in both groups, but only the active IFG-neurofeedback group showed transfer effects (increased IFG activation during transfer session), which correlated with clinical improvements assessed using the CPRS‐R ADHD index scale.
An et al. [25]	China	23 (medication free for >1 month)	32	*M* = 12.09 SD = 1.8	100%	Not reported	Single-blind, counter balanced cross-over, placebo-controlled RCT, comparing MPH to placebo	ReHo	MPH decreased ReHo in the right lingual gyrus and right postcentral gyrus, and increased ReHo in left IFG and right orbitofrontal cortex. ReHo decreases in the right postcentral gyrus and superior parietal lobe following a single dose of MPH were negatively associated with changes in ADHD symptoms at the 8th week, as assessed using the ADHD RS-IV (examined in a subgroup of *N* = 7 with follow-up symptom data).
Criaud et al. [30]	UK	31 (24 receiving stable medication)	0	*M* = 13.90 SD = 1.58	100%	Not reported	2-week real-time fMRI neurofeedback of the right IFG vs. a control para-hippocampal region. Single-blind RCT	Changes in brain activation during error-monitoring associated with right IFC and control neurofeedback	Increases in left insula/IFG/putamen activation during error trials was associated with improvements in ADHD symptoms assessed using the ADHD-RS-IV in the right IFG feedback group.
Cubillo et al. [152]	UK	20 (all medication naïve)	20	Range = 10–17 years old	100%	Not reported	Double-blind, placebo-controlled, crossover RCT comparing single-dose MPH, ATX, and placebo	Task-related brain activation, assessed on and off single doses of MPH and ATX	In the working memory task, drugs increased fronto-temporo-striatal activation and deactivated the default-mode network. However, ATX alone increased and normalized right DLPFC activation, while MPH upregulated left IFG activation.
Cubillo et al. [152]	UK	19 (all medication naïve)	29	Range = 10–17 years old	100%	Not reported	Double-blind, placebo-controlled, crossover RCT comparing single-dose MPH, ATX, and placebo	Task-related brain activation, assessed on and off single doses of MPH and ATX.	During the stop task, both drugs significantly normalized left IFG underactivation observed under placebo. MPH also upregulated and normalized activation in right IFG.
Kowalczyk et al. [75]	UK	14 (all medication naïve)	:27	Range = 10–17 years old	100%	Not reported	Double-blind, placebo-controlled, crossover RCT comparing single-dose MPH, ATX, and placebo	Task-related brain activation, assessed on and off single doses of MPH and ATX	During sustained attention, both drugs enhanced activation of right middle/superior temporal cortex, PCC, and precuneus relative to placebo. Only MPH upregulated left IFG/superior temporal lobe activation.
Liddle et al. [78]	UK	18 (all undergoing MPH treatment)	18	Not reported (9- to 15-year-old range)	Not reported	Not reported	Non-blinded study of chronically medicated MPH responders with ADHD, in which subjects were scanned on and off MPH	Default mode deactivation on and off MPH, assessed during a go/no-go task	MPH normalized default mode deactivation relative to controls.
Lin and Gau [87]	Taiwan	24 (all medication naïve)	24	*M* = 30.32 SD = 9.05	46%	Not reported	8-week double-blind randomized controlled trial comparing ATX against placebo	Changes in resting-state connectivity of key nodes of default mode, affective, dorsal attention, ventral attention, and cognitive control networks	ATX-related improvements in ADHD symptoms were related to pre- to post-treatment changes in functional connectivity, predominantly involving inferior frontal and temporo-parietal regions.
Mizuno et al. [83]	Japan	27 (all medication-free for >5 times medication half-lives)	49	*M* = 10.96, SD = 2.14	100%	Not reported	Double-blind, placebo-controlled, crossover RCT comparing single-dose MPH, and placebo	Dynamic resting-state functional connectivity	Abnormalities in time-varying connectivity observed under placebo were remediated by MPH.
Peterson et al. [21]	USA	16 (all MPH responders)	20	*M* = 13.71 SD = 2.85	69%	94%	Non-blinded study of chronically medicated psychostimulant responders with ADHD, in which subject were scanned on and off MPH	Brain activation was assessed during a stop task both on and off medication	MPH improved deactivation of default mode network in the ADHD group during the stop task.
Rubia et al. [76]	UK	12 (all medication naïve)	12	*M* = 13, SD = 1	100%	Not reported	Double-blind, placebo-controlled, crossover RCT comparing single-dose MPH, and placebo	Task-related brain activation, assessed on and off single doses of MPH	During time discrimination, left IFG/insula and dACC were upregulated by MPH.
Rubia et al. [72]	UK	13 (all medication naïve)	13	*M* = 12.79 SD = 1.5	100%	Not reported	Double-blind, placebo-controlled, crossover RCT comparing single-dose MPH, and placebo	Task-related brain activation, assessed on and off single doses of MPH	MPH upregulated right IFG during sustained attention and vmPFC and caudate during rewarded processing.
Rubia et al. [77]	UK	12 (all medication naïve)	13	*M* = 13, SD = 1	100%	Not reported	Double-blind, placebo-controlled, crossover RCT comparing single-dose MPH, and placebo	Task-related brain activation, assessed on and off single doses of MPH	MPH upregulated right IFG and premotor cortices during the Simon task.
Rubia et al. [153]	UK	12 (all medication naïve)	13	*M* = 13, SD = 1	100%	Not reported	Double-blind, placebo-controlled, crossover RCT comparing single-dose MPH and placebo	Task-related brain activation, assessed on and off single doses of MPH	During error trials on stop task, MPH upregulated bilateral IFG/insula/putamen/caudate and left DLPFC.
Rubia et al. [90]	UK	31 (24 receiving stable medication)	0	*M* = 13.90 SD = 1.58	100%	Not reported	2-week real-time fMRI neurofeedback of the right IFG vs. a control para-hippocampal region. Single-blind RCT.	Changes in functional connectivity assessed during transfer session	Changes in IFG connectivity were specific to the right IFG training group, and correlated with clinical improvements assessed using the CPRS‐R ADHD index scale.
Schrantee et al. [80]	Netherlands	40 children + 48 adults (all medication naïve)	0	Children: 11.5 (0.8); adults: 28.6 (4.6)	100%	Not reported	Subjects scanned before and after a single-dose of MPH	ASL	MPH was associated with reduction widespread cortical CBF reductions in children and adults. CBF reductions within the thalamus were observed only in children.
Shang et al. [88]	Taiwan	38 (all medication naïve)	0	10.5 (2.4)	83%	Not reported	12-week open-label RCT of MPH and ATX	fALFF	Pre- to post-treatment increases in fALFF in the left superior temporal gyrus and left inferior parietal lobule (MPH) and in the left lingual gyrus and left inferior occipital gyrus (ATX) were associated with changes in inattention symptoms. Changes in hyperactivity/impulsivity symptoms were associated with increases in fALFF in the MPH group but decreases in fALFF in the ATX group.
Schulz et al. [89]	USA	36 (13 medication naïve, 8 medicated at enrollment prior to washout)	0	11.2 2.71	83%	Not reported	6 to 8-week MPH and ATX double-blind parallel groups RCT	Brain activation during go/no-go task	Improvement in ADHD symptoms under both drugs was associated with decreased bilateral motor cortex activation. Symptomatic improvement was also related to increased activation following treatment for ATX in right IFG, left ACC, and bilateral posterior cingulate cortex, but decreases in activation in the MPH group.
Silk et al. [79]	Australia	16 (10 medication naïve, 6 withdrawn from meds for 48 hours)	15	M = 13.37 Range = 12.13 to 15.80	100%	Not reported	Double-blind cross-over RCT comparing single doses of MPH, and placebo	Whole-brain resting-state connectivity	MPH was associated with widespread decreases in functional connectivity involving occipital, temporal and subcortical regions.
Smith et al. [74]	UK	20 (all medication naïve)	20	Range = 10–17 years old	100%	Not reported	Double-blind, placebo-controlled, crossover RCT comparing single-dose MPH, ATX and placebo.	Task-related brain activation, assessed on and off single doses of MPH and ATX	Both medications, upregulated right IFG/insula activation during time discrimination. No differences were observed between drugs.
van Elst et al. [71]	Germany	131 at baseline (98 had follow-up data)	0	M = 35.40, SD = 9.8	52%	99%	12-month placebo-controlled RCT of MPH versus placebo	Gray matter volume	MPH was not associated with any significant changes in gray matter volume. Non-significant trends were detected in the cerebellum, which showed increases over time in the MPH group only.
Wang et al. [82]	USA	49 (all medication naïve or medication free for >4 months for children or 12 months for adults)	46	M = 13.52 SD = 5.51	62.1%	Not reported	12-week, placebo-controlled RCT of LDEX versus placebo	Dynamic resting-state functional connectivity	LDEX increased static and decreased dynamic FC. However, decreases in dynamic functional connectivity were associated with the therapeutic effects of LDEX.
Yang et al. [85]	USA	19	0	34.3 (9.3)	68.75%	Not reported	3-week RCT of amphetamine-based stimulant medications	Whole-brain resting-state connectivity	Reductions in connectivity between left DLPFC and bilateral ACC and right insula tracked treatment-related improvement in hyperactive/impulsive symptoms, while reductions in connectivity between bilateral medial frontal and left insula were associated with greater overall improvements in ADHD symptoms.
Yoo et al. [86]	South Korea	20 (all medication-naïve)	27	10.09 (2.5)	74.47%	Not reported	12-week MPH, open label	ALFF, fALFF, resting-state connectivity assessed using ICA dual regression and graph theory measures of resting-state connectivity	Changes in resting-state connectivity and ALFF could explain the 27.1% variance of symptom improvement measured by the K-ARS total score. The strongest predictor was ALFF within bilateral superior parietal lobe.

*ACC* anterior cingulate cortex, *ADHD* attention deficit hyperactivity disorder, *ADHD-RS-IV* ADHD Rating Scale-IV, *ALFF* amplitude of low-frequency fluctuation, *ASL* Arterial spin labeling, *ATX* atomoxetine, *CPRS-r* Conners’ Parent Rating Scale-revised, *dACC* dorsal anterior cingulate cortex, *DICA-IV* Diagnostic Interview for Children and Adolescents – IV, *DLPFC* dorsolateral prefrontal cortex, *fALFF* fractional amplitude of low-frequency fluctuations, *fMRI*; functional magnetic resonance imaging, *IFG* inferior prefrontal gyrus; *ICA* independent component analysis, *K-ARS* Korean ADHD Rating Scale, *LDEX* lisdexamfetamine, *MPH* methylphenidate; *MRI* magnetic resonance imaging, *PCC* posterior cingulate cortex, *RCT* randomized controlled trial, *ReHo* regional homogeneity, *vmPFC* ventromedial prefrontal cortex.

A reasonably large literature, including studies using double-blind placebo-controlled designs in unmedicated youth with ADHD, has examined changes in brain functioning under acute doses of stimulant medication [9, 72–75] (Table 4). Available fMRI studies reported upregulation of cingulo-opercular [9, 73, 76, 77] and striato-thalamic activation [9, 77], and greater default mode deactivation [9, 21, 78] across a range of cognitive tasks. Regarding resting-state fMRI, changes in brain functioning following acute doses have also been reported in cingulo-opercular, striato-thalamic and default mode networks [25, 79–81]. However, results are more varied than those from the task-based literature, arguably reflecting the heterogeneity in processing pipelines, regions of interest, network parcellations and imaging metrics [81]. Nonetheless, two recent studies have converging findings which point to a stabilizing influence of psychostimulants on atypically variable resting-state connectivity patterns in subjects with ADHD, as assessed using dynamic functional connectivity methods [82, 83]. The few studies investigating acute non-stimulant (e.g., ATX) effects have reported largely overlapping patterns of functional brain changes to those seen under single MPH doses [73–75], despite the longer time needed for ATX to produce therapeutic effects and its distinct molecular mechanisms [84].

A further body of work has examined the links between treatment-related improvements in ADHD symptoms and pre- to post-treatment changes in brain functioning over the course of clinical trials. ADHD symptom changes have been associated with changes in functional connectivity between bilateral medial frontal and left insular regions [85] and in fractional amplitude of low-frequency fluctuations (fractional ALFF) within bilateral superior parietal lobe [86]. Studies on ATX suggest that the therapeutic mechanisms of ATX require medium-term changes in the brain [87, 88], and that ATX-related improvements in ADHD symptoms were related to changes in functional connectivity, predominantly involving inferior frontal and temporo-parietal regions [87]. Interestingly, initial studies directly comparing the medium-to-long term effects of MPH and ATX on brain functioning suggest partly different effects [88, 89]. For example, in one study, clinical improvements positively correlated with treatment-related increases in fractional ALFF within left temporo-parietal and bilateral pre- and post-central gyri regions for participants taking MPH, but negatively with changes in fractional ALFF within left occipital lobe and pre- and post-central gyri for patients taking ATX [88]. Regarding task-based fMRI, one medium-term (6–8 weeks) study reported that improvement in symptoms were associated with gains in task-related activation for ATX but reductions in activation for MPH in the right IFG, left anterior cingulate/supplementary motor area, and bilateral posterior cingulate cortex [89].

With regard to non-pharmacological interventions, the aforementioned fMRI neurofeedback trial of the rIFG represents a notable example [30, 90]. The IFG-neurofeedback group showed increases in fronto-striatal activation during an inhibitory control task and in activation and connectivity during a learning transfer test from pre- to post-treatment, which correlated with ADHD symptom improvements [28–30, 90].

Together, these findings provide initial evidence suggesting that distinct neuroimaging biomarkers may be useful for monitoring the longer-term effects of MPH, ATX and right IFG-neurofeedback (fronto-striatal activity/connectivity). Nevertheless, limitations of this body of work include reliance of small sample sizes and lack of replications, as well as more practical limitations of MRI which limit its utility in clinical settings.

### EEG

Several studies have investigated the acute effects of medication on EEG profiles in individuals with ADHD [8] (Table 5). Acute MPH doses decrease theta and alpha power and increase beta power [91, 92] during resting states, suggesting that acute stimulants ameliorate patterns of cortical hypo-arousal associated with ADHD [92]; although non-significant [93, 94] effects have also been reported. Acute MPH administration also increases the amplitude of ERP components often reduced in individuals with ADHD [16, 95], such as P3 [96, 97] and error-related negativity and positivity [98] during go/no-go tasks, whereas effects on N2 amplitudes are more mixed [96, 97]. These partly inconsistent findings likely arise from the use of small, heterogeneous samples and analytic differences (e.g., use of absolute vs. relative power). Initial findings further suggest that acute ATX doses decrease spectral power in delta, theta and beta bands [99].

**Table 5:** Details of EEG studies of candidate monitoring/response biomarkers.

Authors, year	Country	N ADHD	N Control	Age	% Male	% White	Design	Candidate biomarker(s)	Key findings
Aggensteiner et al. [115]	Germany	103 (77 at follow-up)	0	*M* = 8.7, SD = 0.9	85%	Not reported	3-month slow cortical potential-NF vs. semi-active control (electromyogram biofeedback) RCT	Cue P3 and contingent negative variation during cued go/no-go task	Attentional (cue P3) and preparatory (contingent negative variation) brain activity and performance non-specifically reduced after treatment. Contingent negative variation in the slow cortical potential-NF group increased with clinical improvement.
Aldemir et al. [116]	Turkey	20 (all medication free)	20	*M* = 9, SD = 3	Not reported	Not reported	3-month MPH vs. ATX	Power across frequency bands during rest	Similar effects of MPH and ATX on EEG power across frequency bands in the ADHD group, especially at frontal and temporal regions.
Barry et al. [99]	Australia	50 (all medication naïve)	50	*M* = 10.2, SD = 1.5	64%	Not reported	Single dose ATX	Beta power during rest	ATX increased absolute and relative beta power and produced topographic changes in other bands in the ADHD group.
Bresnahan et al. [102]	Australia	50 (DEX responders)	50	*M* = 31, SD = 9	50%	Not reported	4-week DEX, open label	Delta and theta power during rest	Significant reduction in absolute delta, absolute and relative theta, and total power in the ADHD group to levels similar to controls.
Chiarenza et al. [44]	Italy	61 (all medication free for >5 times half-lives)	Not reported (reference database)	*M* = 10.4, SD = 2.9	85%	Not reported	12-month ATX, open label	Absolute power across frequency bands during rest	Treatment-related reductions of absolute power in all frequencies over frontal, central and temporal regions in responders, becoming similar to controls. Non-significant changes in non-responders.
Clarke et al. [104]	Not reported (probably Australia)	50 (all medication naïve or medication free for >5 times half-lives)	40	Range = 8–13	100%	Not reported	6-month MPH or DEX, open label	Theta and beta power, TBR during rest	MPH/DEX increased beta power and reduced theta power and TBR in ADHD group, to levels similar to controls.
Clarke et al. [105]	Not reported (probably Australia)	20 (all inattentive type, medication naïve or medication free for >5 times half-lives)	10	Range = 8–13	100%	Not reported	6-month MPH or DEX, open label	Theta, alpha, beta power, theta/alpha ratio during rest	MPH/DEX increased alpha and beta power and reduced theta power and theta/alpha ratio in inattentive ADHD group, to levels similar to controls.
Groom et al. [96]	UK	28 (all combined type, MPH responders)	28	*M* = 12.5, SD = 1.8	96%	Not reported	Single dose MPH	N2 and P3 amplitudes during go/no-go task	MPH increased N2 and P3 amplitudes in the ADHD group.
Groom et al. [98]	UK	28 (all combined type, MPH responders)	28	*M* = 12.5, SD = 1.8	96%	Not reported	Single dose MPH	ERN and Pe during go/no-go task	MPH increased ERN and Pe amplitudes in the ADHD group.
Hermens et al. [107]	Australia	34 (16 medication naïve, 18 MPH free for >2 weeks)	34	*M* = 13.8, SD = 1.6	82%	Not reported	4-week MPH, open label	Theta power, P3 during oddball task	MPH reduced theta power and increased P3 amplitude in the ADHD group, to levels similar to controls.
Isiten et al. [106]	Turkey	43 (all drug naïve)	0	*M* = 11.9, SD = 2.3	77%	Not reported	1.5-year MPH, open label	Beta power during rest	MPH decreased TBR and increased beta power; no effect on theta power.
Janssen et al. [103]	Netherlands	112 (all medication free for >1 month)	0	*M* = 9.6, SD = 1.6	75%	Not reported	10-week MPH vs. NF vs. physical activity RCT	Theta power during rest and stop signal task	MPH reduced theta power during rest and task more than physical activity. NF reduced theta power during rest more than physical activity. Greater NF-related theta reductions correlated with symptom improvement.
Janssen et al., 2016b [110]	Netherlands	112 (all medication free for >1 month)	0	*M* = 9.6, SD = 1.6	75%	Not reported	10-week MPH vs, NF vs. physical activity RCT	P3 amplitude during stop signal task	MPH increased P3 amplitude more than NF and physical activity. Stimulant-related P3 effects were localized in the thalamus and striatum.
Kratz et al. [117]	Germany	23 (all medication naïve)	0	*M* = 9.0, SD = 1.1	79%	Not reported	8-week MPH vs. ATX, cross-over	Contingent negative variation amplitude during attention network test	MPH but not ATX increased contingent negative variation amplitudes.
Loo et al. [91]	USA	10 (all MPH free for 48 hours)	0	*M* = 10.5, SD = 1.3	80%	Not reported	Single dose MPH vs. placebo, cross-over	Power across frequency bands during rest and CPT	MPH reduced theta and alpha power and increased beta power in responders, with opposite effects in non-responders.
Loo et al. 2004 [92]	USA	36	0	*M* = 10.2, *SD* = 1.3	72%	Not reported	Single dose MPH vs. placebo, cross-over	Power across frequency bands during rest and CPT	MPH increased beta power in responders, with opposite effects in non-responders.
Loo et al. [109]	USA	207 (all medication naïve or medication free for >5 times half-lives)	0	*M* = 10.1, SD = 2.1	68%	83%	8-week MPH, GUAN, MPH + GUAN, RCT	Power across frequency bands during rest	GUAN decreased global alpha power, MPH and MPH + GUAN increased centro-parietal beta power, and MPH + GUAN decreased theta power. Medication-related changes in theta power correlated with behavioral and cognitive improvements.
Lubar et al. [93]	USA	23 (all MPH free for 48 hours)	Not reported (reference database)	Range = 9–11	100%	Not reported	Single dose MPH vs. placebo, cross-over	Coherence, phase and asymmetry during rest	MPH ameliorated atypical EEG coherence, phase and asymmetry patterns in the ADHD group, to levels similar to the reference group.
Luo et al. [56]	China	121	0	*M* = 8.94, range 7.1–12.3	83%	Not reported	3-month remote computerized cognitive, NF, and combined training, RCT	Alpha power during rest	All 3 treatments increased relative alpha power. Pre-training inattention scores corelated negatively with change in relative alpha.
McGough et al. [114]	USA	62 (medication free for >1 month)	0	*M* = 10.4, SD = 1.4	65%	65%	4-week active vs. sham TNS, RCT	Power in delta, theta, beta, gamma frequency bands during rest	TNS increased right-frontal (delta, theta, beta, and gamma) and mid-frontal (gamma) power.
Michelini et al. [118]	USA	207 (all medication naïve or medication free for >5 times half-lives)	0	*M* = 10.1, SD = 2.1	68%	83%	8-week MPH, GUAN, MPH + GUAN, RCT	Event-related mid-occipital power during a Sternberg spatial working memory task with encoding, maintenance and retrieval phases	MPH + GUAN decreased midoccipital theta, alpha and beta across task phases, with significantly greater changes than monotherapies. MPH increased midoccipital theta during retrieval. GUAN produced trend-level reductions in midoccipital alpha during maintenance and retrieval. Treatment-related changes in midoccipital power correlated with ADHD improvements.
Skirrow et al. [108]	UK	41 (all medication free for >1 month [stimulants]) or 6 months [other medication])	48	*M* = 28.5, SD = 9.5	100%	Not reported	3.5-month MPH, open label	Theta power during rest, CPT and sustained attention to response task	MPH normalized in the ADHD group the increase in theta power between rest and task conditions displayed by controls.
Song et al. [94]	South Korea	24	0	*M* = 8.6, SD = 1.4	100%	Not reported	Single dose MPH	Power across frequency bands and TBR during CPT	MPH increased alpha and beta power, decreased theta and delta power, and increased TBR during CPT. No effects during rest.
Verbaten et al. [97]	Netherlands	12	0	*M* = 11.2, SD = 2.1	83%	Not reported	Single dose MPH vs. placebo, RCT	P3 and N2 amplitudes during CPT	MPH increased parietal P3 and frontal N2 amplitudes to targets and non-targets.

*ADHD* attention deficit hyperactivity disorder, *ATX* atomoxetine, *CPT* continuous performance test, *EEG* electroencephalography, *DEX* dexamphetamine, *ERP* event relate potential, *GUAN* guanfacine, *MPH* methylphenidate, *iAPF* individual alpha peak frequency; *M* mean age, *NF* neurofeedback, *RCT* randomized controlled trial; *SD* standard deviation, *TBR* theta/beta ratio, *TNS* trigeminal nerve stimulation.

Studies investigating the longer-term effects of pharmacological treatment on EEG data have generally shown that stimulants ameliorate EEG patterns that differ between individuals with ADHD and neurotypical controls [16, 100–102], albeit often without reaching full normalization. MPH treatment have been shown to increase resting-state beta power and reduce theta power and TBR across several studies [8, 103–106], particularly over frontal and central scalp regions, although non-significant effects have also been reported, [106–109] especially for TBR [8]. Regarding event-related activity, stimulants have consistently shown medium and long-term effects on ERP components associated with impaired sustained attention and cognitive control in individuals with ADHD, particularly for P3 amplitudes [107, 110–113], with P3 increases linked with improvements in clinical and cognitive profiles [111].

Besides EEG studies of stimulants, 12-month ATX treatment produced decreases in EEG resting-state power in delta, theta, and alpha bands in responders but no changes in non-responders [44]. Several studies have also focused on EEG effects of non-pharmacological treatments. Preliminary but encouraging data exist from a 4-week RCT of trigeminal nerve stimulation in children with ADHD, showing that active treatment increased resting state right-frontal (delta, theta, beta, and gamma) and mid-frontal (gamma) spectral power [114]. Several studies have reported effects of neurofeedback, although findings appear quite inconsistent [56, 103, 110, 115]. More generally, the utility of neurofeedback biomarkers remains unclear given the inconclusive findings on the clinical efficacy of neurofeedback [53–55].

Very few EEG studies have compared the effect of different treatments on EEG measures. While similar acute effects of MPH and ATX have been reported on EEG spectral power [116], a cross-over study comparing the effects of 8-week MPH vs. ATX on contingent negative variation amplitudes showed increases with MPH but not ATX [117]. Only one RCT, to our knowledge, compared the long-term effects of stimulant medication (MPH), non-stimulant medication (guanfacine, an alpha-2 agonist) as well as their combination [109, 118]. After 8 weeks, each treatment displayed distinct effects on resting-state spectral power and event-related power during a spatial working memory (WM) task. MPH was associated with increased centro-parietal resting-state beta power and mid-occipital event-related theta power during WM retrieval, which was localized in the primary visual cortex. Guanfacine decreased resting-state alpha power across scalp regions and mid-occipital alpha power during working memory maintenance and retrieval (although event-related findings did not survive multiple-testing corrections). Combined MPH + guanfacine treatment increased centro-parietal resting-state beta power, decreased resting-state theta power across scalp regions, and decreased event-related mid-occipital theta and beta power throughout the WM task, suggesting ameliorating effects on EEG measures showing ADHD-control differences in previous studies [100, 101, 119, 120]. Changes in EEG activity produced by combined medication were also associated with significant clinical and cognitive improvements [109, 118]. Finally, a few notable studies have directly compared the effects of medication and non-pharmacological interventions on EEG measures. In an RCT testing the effects of MPH, neurofeedback and physical activity, the former two treatments showed comparable reductions in theta power and smaller reductions with physical activity [103]. Yet, only MPH increased P3 amplitudes during a go/no-go task compared with neurofeedback and physical activity [110]. These differences were no longer evident after 6 months, suggesting similar long-term effects of these treatments [121].

Overall, findings reviewed here suggests that EEG profiles, particularly event-related measures (e.g., P3 amplitudes and event-related power modulations), are promising monitoring biomarkers. Future studies should replicate these encouraging findings in more heterogeneous samples and test the effects of multiple treatments. The application of source localization techniques is particularly promising for uncovering underlying brain mechanisms of EEG biomarkers and may also guide the development of novel non-pharmacological treatments [1].

## Discussion

In this article we have reviewed progress in the discovery of treatment biomarkers for ADHD and their translation towards personalized treatment approaches, with a particular focus on predictive and monitoring/response biomarkers. Several pre-treatment profiles have been shown to predict response to pharmacological treatments for ADHD, with more preliminary but encouraging findings for response to non-pharmacological treatments. The most promising measures for treatment prediction are EEG measures such as iAPF and event-related beta power modulations, and genetic markers involved in PRS and CNVs, whereas the MRI literature has generally yielded more mixed findings largely based on small samples. These pre-treatment profiles represent candidate predictive biomarkers which, in the future, may assist in the stratification of patients with ADHD to specific treatments. Research leveraging repeated biomarker assessments further suggests that clinical response to ADHD treatments is underpinned by treatment-specific changes in brain profiles. These treatment-related changes have typically been consistent with the amelioration of neural patterns that are altered in children and adults with ADHD, although some evidence of “better than typical” post-treatment profiles have also been reported, suggesting potential compensatory mechanisms [118, 122]. These findings point to promising candidate monitoring/response biomarkers which may not only assist in future monitoring of treatment response, but also guide development of novel treatments (e.g., neurotherapeutics [11]) targeting neurobiological mechanisms.

Overall, this body of research represents a solid research base for the development of biomarker approaches and for the future allocation of patients to existing and novel pharmacological and non-pharmacological treatments based on their individual behavioral and neurobiological profiles, consistent with the principles of precision and personalized medicine [26, 122, 123]. Nevertheless, despite this considerable progress, the available literature does not yet provide sufficiently strong evidence for actionable treatment biomarkers for ADHD in clinical settings. Biomarker studies have provided quite heterogenous findings to date, likely due to limitations of study samples, study designs and analysis methods used [3, 81], as well as the known heterogeneity of the ADHD population [10, 124]. In the following paragraphs, we highlight key directions for future research studies and methodological and practical changes required to facilitate the clinical translation of treatment biomarkers for ADHD, with the goal of promoting equitable access to personalized medicine practices for all treatment-seeking individuals.

First, there is a widespread lack of replication and out-of-sample validation of findings for most candidate predictive and monitoring/response biomarkers, with only a few notable exceptions [52]. Most studies have used small samples (e.g., *N* < 100 participants for MRI/EEG studies), which are unlikely to allow for reliable estimates of the associations of genetic and brain biomarkers with measures of clinical effectiveness [125, 126]. The field needs to move towards systematic replication and out-of-sample validation of promising biomarker findings in larger samples, which will be essential to guarantee that the validity of biomarkers will generalize to individuals in clinical settings in the future. This is likely to require collaborative, multi-site recruitment efforts, following examples set by biomarker research on other neurodevelopmental and psychiatric disorders [127, 128].

Second, alongside increasing sample sizes and validation efforts, future research will be needed to increase the diversity of samples with regard to age, sex, comorbidities, ethnicity, race, and geographical region. As highlighted throughout our review, most evidence to date is based on samples of children (mainly boys) from White majority backgrounds and high-income countries, typically with few psychiatric and medical co-occurring conditions, even though comorbidities are the norm in patients with ADHD [6, 129]. Several reviewed studies conducted in Western countries do not even provide information on race/ethnicity (Tables 1–5), likely suggesting the use of all-White or predominantly-White samples that are not representative of the wider population of individuals with ADHD [130]. While underrepresented minority populations have largely been excluded from biomarker research across methodologies, this issue is particularly acute for genetic research, as nearly all studies reviewed here have been conducted on White and non-Hispanic populations [131–134]. In parallel, future efforts should also increase the cultural competence of clinicians responsible for clinical assessments and treatment decisions. [135] Further, considering the clinical and etiological heterogeneity that characterizes the ADHD population [10, 124], different subgroups of individuals may display different degrees of clinical improvements and neurobiological changes in response to any given treatment. Studies investigating biomarkers of narrow aspects of ADHD symptomatology (e.g., inattentive symptoms), focusing on specific subgroups of the ADHD population (e.g., children with co-occurring ADHD and autism), and delineating data-driven clusters of individuals with ADHD will be especially useful to address this issue. The use of more heterogeneous and diverse samples and employment of inclusive research practices will be essential to ensure equitable access to future clinical applications of treatment biomarkers for people with ADHD.

Third, most studies have examined associations between specific biomarkers and treatment outcomes using correlational and group-level analyses, but any biomarker is likely to individually explain only a small amount of variance in treatment response. Classifiers and machine learning algorithms are becoming particularly popular approaches to handle high dimensionality and maximize predictive accuracy of biomarkers [3, 136]. The few studies that adopted these multivariate methods to predict treatment response at the level of individual subjects have used small sample sizes, which are known to be highly sensitive to overfitting [20, 57, 137]. Moreover, most studies did not examine whether biomarker profiles explain clinically useful variance over and above routinely-collected information such as baseline ADHD symptoms, comorbidities and neuropsychological functioning. Future studies will need to use multivariate approaches to aggregate several biomarkers across multiple units of analysis as well as clinical and demographic characteristics, in order to establish the clinical utility of promising biomarkers in combination with other more readily-available information. These combined biomarkers will then need to be tested at the individual level in independent samples, using metrics such as AUC, sensitivity, specificity, negative and positive predictive values.

Fourth, although a key aim of personalized medicine is to determine which treatments may work best out of the available options for a given patient [26, 122, 123], little work has examined the treatment-specificity of associations between brain pattern and treatment outcomes. Future research testing treatment-specific associations will be crucial for developing mechanistic models of ADHD treatments and individually tailored treatment algorithms. Such studies would also be particularly valuable to define the precise mechanisms of action of current treatment, which could then be targeted in the development of novel treatments, such as neurostimulation of the circuits sensitive to the effects of current medications. In addition, existing studies have mainly focused on associations with treatment response measured as improvements in ADHD symptoms and impairment, and more work is needed to examine whether individual differences in adverse side effects and medication tolerability can be predicted and monitored using biomarkers. Finally, quantifying biomarker thresholds for inclusion criterion or stratification of participants to treatment and subsequent testing in independent samples is a critical next step in deploying biomarkers in clinical practice and personalized medicine.

Fifth, since most of the available studies have conducted biomarker and clinical assessments only over a few weeks or months, we know very little about the clinical utility of biomarkers over longer time periods. Longitudinal work following patients with ADHD over multiple years has shown that remission from ADHD symptoms follows a highly non-linear trajectory for most patients [138], and it is not uncommon for the effects of medication to decrease or for side effects to emerge over time, despite high initial rates of response and tolerability [139, 140]. Predictive biomarkers derived before treatment allocation may thus not be indicative of longer-term treatment success [138], and future studies using longer time frames of several months or years are needed. Similarly, with regard to monitoring/response biomarkers, while most studies have focused on the therapeutic brain mechanisms of ADHD treatments at the group level, particularly with stimulants, more work is needed to explore whether individual differences in these changes act as a source of variation in treatment response. Potential clinical applications may be better informed by knowledge of how medium-term changes in biomarkers relate to treatment responses over the longer term in ADHD [138]. Thus, future studies will need to examine whether changes in brain functioning over the initial weeks of treatment predict treatment response over the longer-term, beyond the timeframe of previous studies.

Sixth, very limited work has sought to establish the reliability, robustness and reproducibility of ADHD treatment biomarkers, although these are essential biomarker characteristics that are critical for future clinical application [3, 141, 142]. The reliability of candidate neuroimaging and EEG measures has only recently been systematically evaluated [126, 143–145]. Findings indicate that while the reliability of certain EEG measures (particularly resting state spectral power) tends to be good in 5–10 min recording conditions [119, 146, 147], the reliability of fMRI measures becomes acceptable only with longer (i.e., over 15–20 min) recordings [145, 148]. In addition, given the flexibility in neuroimaging processing and analysis methods and the resultant large number of researcher degrees of freedom, the pre-registration of future data processing and analysis plans is essential to limit type-I errors and inflated effect sizes known to effect non-registered studies [149], as are multiverse studies in which the robustness of study findings are examined across multiple distinct pipelines and analysis strategies [150].

Finally, from a practical perspective, any neuroimaging, EEG, and genetic biomarker findings must not only be generalizable across patient subpopulations, but also across treatment sites and various testing equipment and procedures. The development of standardized biomarker testing protocols and training of healthcare professionals responsible for biomarker testing will be important challenges to face prior to clinical implementation. Further, the cost-effectiveness of brain and genetic markers is also yet to be determined from a health economics perspective, and will require pragmatic RCTs comparing the effectiveness and costs of biomarkers approaches vs. care as usual [122]. These cost-benefit considerations will be particularly salient for neuroimaging biomarkers, considering the high cost of data collection. Importantly, future efforts should also place particular attention to the acceptability of biomarker approaches for people with ADHD (i.e., participatory research and research co-production approaches [151]). This is because even the most predictive and reliable biomarker will have limited clinical utility if most patients were unwilling to undergo testing necessary to derive the biomarker. This will be especially important for biomarkers requiring more involved assessments (e.g., MRI) and measures with important ethical implications (e.g., genetics). Inclusive and participatory research practices will be crucial to maximize the feasibility and uptake of future clinical applications of treatment biomarkers by diverse groups of individuals with ADHD worldwide.

In conclusion, this article sought to highlight progress in the development of treatment biomarkers for ADHD and set clear guidance for future studies to move the field forward. Whereas most of the available research on ADHD biomarker to date has taken a primarily mechanistic approach, we hope that our recommendations will contribute to a shift toward more translational and clinically useful approaches, as well as more robust methodologies, and larger and more diverse samples. We believe that these steps will be essential to promote clinical translation of biomarker research and enhance personalized treatment decisions for diverse groups of individuals with ADHD.

## References

[1] Loo SK, Lenartowicz A, Makeig S. Research Review: use of EEG biomarkers in child psychiatry research - current state and future directions. J Child Psychol Psychiatry. 2015. 10.1111/jcpp.12435. 10.1111/jcpp.12435 PMC4689673 26099166

[2] Abi-Dargham A, Horga G (2016). The search for imaging biomarkers in psychiatric disorders. Nat Med.

[3] Uddin LQ, Dajani DR, Voorhies W, Bednarz H, Kana RK (2017). Progress and roadblocks in the search for brain-based biomarkers of autism and attention-deficit/hyperactivity disorder. Transl Psychiatry.

[4] Cuthbert BN, Insel TR (2013). Toward the future of psychiatric diagnosis: the seven pillars of RDoC. BMC Med.

[5] Michelini G, Palumbo IM, DeYoung CG, Latzman RD, Kotov R (2021). Linking RDoC and HiTOP: a new interface for advancing psychiatric nosology and neuroscience. Clin Psychol Rev.

[6] Franke B, Michelini G, Asherson P, Banaschewski T, Bilbow A, Buitelaar JK (2018). Live fast, die young? A review on the developmental trajectories of ADHD across the lifespan. Eur Neuropsychopharmacol.

[7] Sudre G, Mangalmurti A, Shaw P (2018). Growing out of attention deficit hyperactivity disorder: Insights from the ‘remitted’ brain. Neurosci Biobehav Rev.

[8] Kirkland AE, Holton KF (2019). Measuring treatment response in pharmacological and lifestyle interventions using electroencephalography in ADHD: a review. Clin EEG Neurosci.

[9] Rubia K, Alegria AA, Cubillo AI, Smith AB, Brammer MJ, Radua J (2014). Effects of stimulants on brain function in attention-deficit/hyperactivity disorder: a systematic review and meta-analysis. Biol Psychiatry.

[10] Nigg JT, Sibley MH, Thapar A, Karalunas SL (2020). Development of ADHD: etiology, heterogeneity, and early life course. Annu Rev Dev Psychol.

[11] Rubia K, Westwood S, Aggensteiner P-M, Brandeis D (2021). Neurotherapeutics for attention deficit/hyperactivity disorder (ADHD): a review. Cells.

[12] Group BDW (2001). Biomarkers and surrogate endpoints: preferred definitions and conceptual framework. Clin Pharmacol Ther.

[13] FDA-NIH Biomarker Working Group. BEST (Biomarkers, EndpointS, and other Tools) Resource. Food and Drug Administration (US): Silver Spring (MD); 2016. http://www.ncbi.nlm.nih.gov/books/NBK326791/ (accessed 23 May 2020). 27010052

[14] Shephard E, Zuccolo PF, Idrees I, Godoy PBG, Salomone E, Ferrante C (2022). Systematic review and meta-analysis: the science of early-life precursors and interventions for attention-deficit/hyperactivity disorder. J Am Acad Child Adolesc Psychiatry.

[15] Pereira-Sanchez V, Castellanos FX (2021). Neuroimaging in attention-deficit/hyperactivity disorder. Curr Opin Psychiatry.

[16] Kaiser A, Aggensteiner P-M, Baumeister S, Holz NE, Banaschewski T, Brandeis D (2020). Earlier versus later cognitive event-related potentials (ERPs) in attention-deficit/hyperactivity disorder (ADHD): a meta-analysis. Neurosci Biobehav Rev.

[17] Olbrich S, van Dinteren R, Arns M (2015). Personalized medicine: review and perspectives of promising baseline EEG biomarkers in major depressive disorder and attention deficit hyperactivity disorder. Neuropsychobiology.

[18] Kim J-S, Lee KH, Hwang C-S, Kim J-W (2022). Subcortical volumetric alterations as potential predictors of methylphenidate treatment response in youth with attention-deficit/hyperactivity disorder. J Psychiatry Neurosci.

[19] Moreno A, Duno L, Hoekzema E, Picado M, Martin LM, Fauquet J (2014). Striatal volume deficits in children with ADHD who present a poor response to methylphenidate. Eur Child Adolesc Psychiatry.

[20] Griffiths KR, Braund TA, Kohn MR, Clarke S, Williams LM, Korgaonkar MS (2021). Structural brain network topology underpinning ADHD and response to methylphenidate treatment. Transl Psychiatry.

[21] Peterson BS, Potenza MN, Wang Z, Zhu H, Martin A, Marsh R (2009). An FMRI study of the effects of psychostimulants on default-mode processing during Stroop task performance in youths with ADHD. Am J Psychiatry.

[22] Norman LJ, Sudre G, Bouyssi-Kobar M, Sharp W, Shaw P (2021). A longitudinal study of resting-state connectivity and response to psychostimulant treatment in ADHD. Am J Psychiatry.

[23] Fusar-Poli P, Rubia K, Rossi G, Sartori G, Balottin U (2012). Striatal dopamine transporter alterations in ADHD: pathophysiology or adaptation to psychostimulants? A meta-analysis. Am J Psychiatry.

[24] Hong S-B, Harrison BJ, Fornito A, Sohn C-H, Song I-C, Kim J-W (2015). Functional dysconnectivity of corticostriatal circuitry and differential response to methylphenidate in youth with attention-deficit/hyperactivity disorder. J Psychiatry Neurosci.

[25] An L, Cao X-H, Cao Q-J, Sun L, Yang L, Zou Q-H (2013). Methylphenidate normalizes resting-state brain dysfunction in boys with attention deficit hyperactivity disorder. Neuropsychopharmacology.

[26] Arns M, van Dijk H, Luykx JJ, van Wingen G, Olbrich S (2021). Stratified psychiatry: tomorrow’s precision psychiatry?. Eur Neuropsychopharmacol.

[27] Schulz KP, Bédard A-CV, Fan J, Hildebrandt TB, Stein MA, Ivanov I (2017). Striatal activation predicts differential therapeutic responses to methylphenidate and atomoxetine. J Am Acad Child Adolesc Psychiatry.

[28] Alegria AA, Wulff M, Brinson H, Barker GJ, Norman LJ, Brandeis D (2017). Real‐time f MRI neurofeedback in adolescents with attention deficit hyperactivity disorder. Hum Brain Mapp.

[29] Lam S-L, Criaud M, Alegria A, Barker GJ, Giampietro V, Rubia K (2020). Neurofunctional and behavioural measures associated with fMRI-neurofeedback learning in adolescents with Attention-Deficit/Hyperactivity Disorder. NeuroImage Clin.

[30] Criaud M, Wulff M, Alegria AA, Barker GJ, Giampietro V, Rubia K (2020). Increased left inferior fronto-striatal activation during error monitoring after fMRI neurofeedback of right inferior frontal cortex in adolescents with attention deficit hyperactivity disorder. NeuroImage Clin.

[31] Lukito S, Norman L, Carlisi C, Radua J, Hart H, Simonoff E (2020). Comparative meta-analyses of brain structural and functional abnormalities during cognitive control in attention-deficit/hyperactivity disorder and autism spectrum disorder. Psychol Med.

[32] Norman LJ, Carlisi C, Lukito S, Hart H, Mataix-Cols D, Radua J (2016). Structural and functional brain abnormalities in attention-deficit/hyperactivity disorder and obsessive-compulsive disorder: a comparative meta-analysis. JAMA Psychiatry.

[33] Loo SK, Makeig S (2012). Clinical utility of EEG in attention-deficit/hyperactivity disorder: a research update. Neurotherapeutics.

[34] Jeste SS, Frohlich J, Loo SK (2015). Electrophysiological biomarkers of diagnosis and outcome in neurodevelopmental disorders. Curr Opin Neurol.

[35] Sari Gokten E, Tulay EE, Beser B, Elagoz Yuksel M, Arikan K, Tarhan N (2019). Predictive Value of Slow and Fast EEG Oscillations for Methylphenidate Response in ADHD. Clin EEG Neurosci.

[36] Ogrim G, Hestad KA, Kropotov J, Sandvik L, Candrian G, Brunner JF. Predicting the clinical outcome of stimulant medication in pediatric attention-deficit/hyperactivity disorder: data from quantitative electroencephalography, event-related potentials, and a go/no-go test. Neuropsychiatr Dis Treat. 2014;10:231–42. 10.2147/NDT.S56600 PMC3921081 24523588

[37] Bellato A, Arora I, Hollis C, Groom MJ (2020). Is autonomic nervous system function atypical in attention deficit hyperactivity disorder (ADHD)? A systematic review of the evidence. Neurosci Biobehav Rev.

[38] Arns M, Vollebregt MA, Palmer D, Spooner C, Gordon E, Kohn M (2018). Electroencephalographic biomarkers as predictors of methylphenidate response in attention-deficit/hyperactivity disorder. Eur Neuropsychopharmacol.

[39] Arns M, Conners CK, Kraemer HC (2013). A decade of EEG theta/beta ratio research in ADHD: a meta-analysis. J Atten Disord.

[40] Arns M, Loo SK, Sterman MB, Heinrich H, Kuntsi J, Asherson P (2016). Editorial perspective: how should child psychologists and psychiatrists interpret FDA device approval? Caveat emptor. J Child Psychol Psychiatr.

[41] Sangal RB, Sangal JM (2006). Attention-deficit/hyperactivity disorder: use of cognitive evoked potential (P300) to predict treatment response. Clin Neurophysiol.

[42] Ogrim G, Kropotov JD (2019). Predicting clinical gains and side effects of stimulant medication in pediatric attention-deficit/hyperactivity disorder by combining measures From qEEG and ERPs in a cued GO/NOGO task. Clin EEG Neurosci.

[43] Young ES, Perros P, Price GW, Sadler T (1995). Acute challenge ERP as a prognostic of stimulant therapy outcome in attention-deficit hyperactivity disorder. Biol Psychiatry.

[44] Chiarenza GA, Chabot R, Isenhart R, Montaldi L, Chiarenza MP, Torto MGL (2016). The quantified EEG characteristics of responders and non-responders to long-term treatment with atomoxetine in children with attention deficit hyperactivity disorders. Int J Psychophysiol.

[45] Griffiths KR, Jurigova BG, Leikauf JE, Palmer D, Clarke SD, Tsang TW (2019). A signature of attention-elicited electrocortical activity distinguishes response from non-response to the non-stimulant atomoxetine in children and adolescents with ADHD. J Atten Disord.

[46] Sangal RB, Sangal JM (2005). Attention-deficit/hyperactivity disorder: cognitive evoked potential (P300) amplitude predicts treatment response to atomoxetine. Clin Neurophysiol.

[47] Leuchter AF, McGough JJ, Korb AS, Hunter AM, Glaser PEA, Deldar A (2014). Neurophysiologic predictors of response to atomoxetine in young adults with attention deficit hyperactivity disorder: a pilot project. J Psychiatr Res.

[48] Singh G, Arun P, Das S, Kaur D (2021). Can EEG predict response to atomoxetine in attention deficit hyperactivity disorder at 1 Week?. J Atten Disord.

[49] Loo SK, Salgari GC, Ellis A, Cowen J, Dillon A, McGough JJ (2021). Trigeminal nerve stimulation for attention-deficit/hyperactivity disorder: cognitive and electroencephalographic predictors of treatment response. J Am Acad Child Adolesc Psychiatry.

[50] Krepel N, Egtberts T, Sack AT, Heinrich H, Ryan M, Arns M (2020). A multicenter effectiveness trial of QEEG-informed neurofeedback in ADHD: Replication and treatment prediction. Neuroimage Clin.

[51] Bazanova OM, Auer T, Sapina EA (2018). On the efficiency of individualized theta/beta ratio neurofeedback combined with forehead EMG training in ADHD Children. Front Hum Neurosci.

[52] Voetterl H, van Wingen G, Michelini G, Griffiths KR, Gordon E, DeBeus R (2022). Brainmarker-I differentially predicts remission to various attention-deficit/hyperactivity disorder treatments: a discovery, transfer, and blinded validation study. Biol Psychiatry Cogn Neurosci Neuroimaging.

[53] Schönenberg M, Wiedemann E, Schneidt A, Scheeff J, Logemann A, Keune PM (2017). Neurofeedback, sham neurofeedback, and cognitive-behavioural group therapy in adults with attention-deficit hyperactivity disorder: a triple-blind, randomised, controlled trial. Lancet Psychiatry.

[54] Van Doren J, Arns M, Heinrich H, Vollebregt MA, Strehl U, Loo K (2019). Sustained effects of neurofeedback in ADHD: a systematic review and meta-analysis. Eur Child Adolesc Psychiatry.

[55] Neurofeedback Collaborative Group. Double-Blind Placebo-Controlled Randomized Clinical Trial of Neurofeedback for Attention-Deficit/Hyperactivity Disorder With 13-Month Follow-up. J Am Acad Child Adolesc Psychiatry. 2020. 10.1016/j.jaac.2020.07.906. 10.1016/j.jaac.2020.07.906 PMC7904968 32853703

[56] Luo X, Guo X, Zhao Q, Zhu Y, Chen Y, Zhang D, et al. A randomized controlled study of remote computerized cognitive, neurofeedback, and combined training in the treatment of children with attention-deficit/hyperactivity disorder. Eur Child Adolesc Psychiatry. 2022. 10.1007/s00787-022-01956-1. 10.1007/s00787-022-01956-1 PMC8857637 35182242

[57] Michelini G, Lenartowicz A, Vera JD, Bilder RM, McGough JJ, McCracken JT (2022). Electrophysiological and clinical predictors of methylphenidate, guanfacine, and combined treatment outcomes in children with ADHD. J Am Acad Child Adolesc Psychiatry.

[58] Arns M, Gunkelman J, Breteler M, Spronk D (2008). EEG phenotypes predict treatment outcome to stimulants in children with ADHD. J Integr Neurosci.

[59] Gul MK, Sener EF, Onal MG, Demirci E (2022). Role of the norepinephrine transporter polymorphisms in atomoxetine treatment: From response to side effects in children with ADHD. J Psychopharmacol.

[60] Myer NM, Boland JR, Faraone SV (2018). Pharmacogenetics predictors of methylphenidate efficacy in childhood ADHD. Mol Psychiatry.

[61] Kendler KS (2013). What psychiatric genetics has taught us about the nature of psychiatric illness and what is left to learn. Mol Psychiatry.

[62] Pagerols M, Richarte V, Sánchez-Mora C, Rovira P, Soler Artigas M, Garcia-Martínez I (2018). Integrative genomic analysis of methylphenidate response in attention-deficit/hyperactivity disorder. Sci Rep.

[63] Zhong Y, Yang B, Su Y, Qian Y, Cao Q, Chang S (2020). The association with quantitative response to attention-deficit/hyperactivity disorder medication of the previously identified neurodevelopmental network genes. J Child Adolesc Psychopharmacol.

[64] Brikell I, Wimberley T, Albiñana C, Pedersen EM, Vilhjálmsson BJ, Agerbo E, et al. Genetic, clinical, and sociodemographic factors associated with stimulant treatment outcomes in ADHD. Am J Psychiatry 2021; 10.1176/appi.ajp.2020.20121686. 10.1176/appi.ajp.2020.20121686 PMC10951468 34154395

[65] Elia J, Glessner JT, Wang K, Takahashi N, Shtir CJ, Hadley D (2011). Genome-wide copy number variation study associates metabotropic glutamate receptor gene networks with attention deficit hyperactivity disorder. Nat Genet.

[66] Elia J, Ungal G, Kao C, Ambrosini A, De Jesus-Rosario N, Larsen L (2018). Fasoracetam in adolescents with ADHD and glutamatergic gene network variants disrupting mGluR neurotransmitter signaling. Nat Commun.

[67] Hegvik T-A, Waløen K, Pandey SK, Faraone SV, Haavik J, Zayats T (2021). Druggable genome in attention deficit/hyperactivity disorder and its co-morbid conditions. New avenues for treatment. Mol Psychiatry.

[68] Yu G, Li G-F, Markowitz JS (2016). Atomoxetine: a review of its pharmacokinetics and pharmacogenomics relative to drug disposition. J Child Adolesc Psychopharmacol.

[69] Bouziane C, Filatova OG, Schrantee A, Caan MW, Vos FM, Reneman L (2019). White matter by diffusion MRI following methylphenidate treatment: a randomized control trial in males with attention-deficit/hyperactivity disorder. Radiology.

[70] Walhovd KB, Amlien I, Schrantee A, Rohani DA, Groote I, Bjørnerud A (2020). Methylphenidate effects on cortical thickness in children and adults with attention-deficit/hyperactivity disorder: a randomized clinical trial. Am J Neuroradiol.

[71] van Elst LT, Maier S, Klöppel S, Graf E, Killius C, Rump M (2016). The effect of methylphenidate intake on brain structure in adults with ADHD in a placebo-controlled randomized trial. J Psychiatry Neurosci.

[72] Rubia K, Halari R, Cubillo A, Mohammad A-M, Brammer M, Taylor E (2009). Methylphenidate normalises activation and functional connectivity deficits in attention and motivation networks in medication-naive children with ADHD during a rewarded continuous performance task. Neuropharmacology.

[73] Cubillo A, Smith AB, Barrett N, Giampietro V, Brammer MJ, Simmons A (2014). Shared and drug-specific effects of atomoxetine and methylphenidate on inhibitory brain dysfunction in medication-naive ADHD boys. Cereb Cortex.

[74] Smith A, Cubillo A, Barrett N, Giampietro V, Simmons A, Brammer M (2013). Neurofunctional effects of methylphenidate and atomoxetine in boys with attention-deficit/hyperactivity disorder during time discrimination. Biol Psychiatry.

[75] Kowalczyk OS, Cubillo AI, Smith A, Barrett N, Giampietro V, Brammer M (2019). Methylphenidate and atomoxetine normalise fronto-parietal underactivation during sustained attention in ADHD adolescents. Eur Neuropsychopharmacol.

[76] Rubia K, Halari R, Christakou A, Taylor E (2009). Impulsiveness as a timing disturbance: neurocognitive abnormalities in attention-deficit hyperactivity disorder during temporal processes and normalization with methylphenidate. Philos Trans R Soc B Biol Sci.

[77] Rubia K, Halari R, Cubillo A, Smith AB, Mohammad A-M, Brammer M (2011). Methylphenidate normalizes fronto-striatal underactivation during interference inhibition in medication-naive boys with attention-deficit hyperactivity disorder. Neuropsychopharmacol.

[78] Liddle EB, Hollis C, Batty MJ, Groom MJ, Totman JJ, Liotti M (2011). Task‐related default mode network modulation and inhibitory control in ADHD: Effects of motivation and methylphenidate. J Child Psychol Psychiatry.

[79] Silk TJ, Malpas C, Vance A, Bellgrove MA (2017). The effect of single-dose methylphenidate on resting-state network functional connectivity in ADHD. Brain imaging behav.

[80] Schrantee A, Mutsaerts H, Bouziane C, Tamminga HGH, Bottelier MA, Reneman L (2017). The age-dependent effects of a single-dose methylphenidate challenge on cerebral perfusion in patients with attention-deficit/hyperactivity disorder. NeuroImage Clin.

[81] Pereira-Sanchez V, Franco AR, Vieira D, de Castro-Manglano P, Soutullo C, Milham MP (2021). Systematic review: medication effects on brain intrinsic functional connectivity in patients with attention-deficit/hyperactivity disorder. J Am Acad Child Adolesc Psychiatry.

[82] Wang Y, Kessel E, Lee S, Hong S, Raffanello E, Hulvershorn LA, et al. Causal effects of psychostimulants on neural connectivity: a mechanistic, randomized clinical trial. J Child Psychol Psychiatry. 2022. 10.1111/jcpp.13585. 10.1111/jcpp.13585 PMC9360200 35141898

[83] Mizuno Y, Cai W, Supekar K, Makita K, Takiguchi S, Tomoda A, et al. Methylphenidate remediates aberrant brain network dynamics in children with attention-deficit/hyperactivity disorder: a randomized controlled trial. NeuroImage. 2022;257:119332. 10.1016/j.neuroimage.2022.119332 PMC9286726 35640787

[84] Cortese S, Aoki YY, Itahashi T, Castellanos FX, Eickhoff SB. Systematic review and meta-analysis: resting state functional magnetic resonance imaging studies of attention-deficit/hyperactivity disorder. J Am Acad Child Adolesc Psychiatry. 2021;60:61–75. 10.1016/j.jaac.2020.08.014 32946973

[85] Yang Z, Kelly C, Castellanos FX, Leon T, Milham MP, Adler LA (2016). Neural correlates of symptom improvement following stimulant treatment in adults with attention-deficit/hyperactivity disorder. J Child Adolesc Psychopharmacol.

[86] Yoo JH, Kim D, Choi J, Jeong B (2018). Treatment effect of methylphenidate on intrinsic functional brain network in medication-naïve ADHD children: a multivariate analysis. Brain Imaging Behav.

[87] Lin H-Y, Gau SS-F. Atomoxetine treatment strengthens an anti-correlated relationship between functional brain networks in medication-naïve adults with attention-deficit hyperactivity disorder: a randomized double-blind placebo-controlled clinical trial. Int J Neuropsychopharmacol. 2016;19:pyv094. 10.1093/ijnp/pyv094 PMC4815465 26377368

[88] Shang CY, Yan CG, Lin HY, Tseng WY, Castellanos FX, Gau SS (2016). Differential effects of methylphenidate and atomoxetine on intrinsic brain activity in children with attention deficit hyperactivity disorder. Psychol Med.

[89] Schulz KP, Fan J, Bédard A-CV, Clerkin SM, Ivanov I, Tang CY (2012). Common and unique therapeutic mechanisms of stimulant and nonstimulant treatments for attention-deficit/hyperactivity disorder. Arch Gen Psychiatry.

[90] Rubia K, Criaud M, Wulff M, Alegria A, Brinson H, Barker G (2019). Functional connectivity changes associated with fMRI neurofeedback of right inferior frontal cortex in adolescents with ADHD. NeuroImage.

[91] Loo SK, Teale PD, Reite ML (1999). EEG correlates of methylphenidate response among children with ADHD: a preliminary report. Biol Psychiatry.

[92] Loo SK, Hopfer C, Teale PD, Reite ML (2004). EEG correlates of methylphenidate response in ADHD: association with cognitive and behavioral measures. J Clin Neurophysiol.

[93] Lubar JF, White JN, Swartwood MO, Swartwood JN (1999). Methylphenidate effects on global and complex measures of EEG. Pediatr Neurol.

[94] Song DH, Shin DW, Jon DI, Ha EH (2005). Effects of methylphenidate on quantitative EEG of boys with attention-deficit hyperactivity disorder in continuous performance test. Yonsei Med J.

[95] Michelini G, Kitsune GL, Cheung CH, Brandeis D, Banaschewski T, Asherson P (2016). Attention-deficit/hyperactivity disorder remission is linked to better neurophysiological error detection and attention-vigilance processes. Biol Psychiatry.

[96] Groom MJ, Scerif G, Liddle PF, Batty MJ, Liddle EB, Roberts KL (2010). Effects of motivation and medication on electrophysiological markers of response inhibition in children with attention-deficit/hyperactivity disorder. Biol Psychiatry.

[97] Verbaten MN, Overtoom CC, Koelega HS, Swaab-Barneveld H, van der Gaag RJ, Buitelaar J (1994). Methylphenidate influences on both early and late ERP waves of ADHD children in a continuous performance test. J Abnorm Child Psychol.

[98] Groom MJ, Liddle EB, Scerif G, Liddle PF, Batty MJ, Liotti M (2013). Motivational incentives and methylphenidate enhance electrophysiological correlates of error monitoring in children with attention deficit/hyperactivity disorder. J Child Psychol Psychiatry.

[99] Barry RJ, Clarke AR, Hajos M, McCarthy R, Selikowitz M, Bruggemann JM (2009). Acute atomoxetine effects on the EEG of children with attention-deficit/hyperactivity disorder. Neuropharmacol.

[100] Clarke AR, Barry RJ, Johnstone S (2020). Resting state EEG power research in attention-deficit/hyperactivity disorder: a review update. Clin Neurophysiol.

[101] Michelini G, Salmastyan G, Vera JD, Lenartowicz A (2022). Event-related brain oscillations in attention-deficit/hyperactivity disorder (ADHD): a systematic review and meta-analysis. Int J Psychophysiol.

[102] Bresnahan SM, Barry RJ, Clarke AR, Johnstone SJ (2006). Quantitative EEG analysis in dexamphetamine-responsive adults with attention-deficit/hyperactivity disorder. Psychiatry Res.

[103] Janssen TWP, Bink M, Geladé K, van Mourik R, Maras A, Oosterlaan J (2016). A randomized controlled trial into the effects of neurofeedback, methylphenidate, and physical activity on EEG power spectra in children with ADHD. J Child Psychol Psychiatry.

[104] Clarke AR, Barry RJ, Bond D, McCarthy R, Selikowitz M (2002). Effects of stimulant medications on the EEG of children with attention-deficit/hyperactivity disorder. Psychopharmacology.

[105] Clarke AR, Barry RJ, McCarthy R, Selikowitz M, Brown CR, Croft RJ (2003). Effects of stimulant medications on the EEG of children with attention-deficit/hyperactivity disorder predominantly inattentive type. Int J Psychophysiol.

[106] Isiten HN, Cebi M, Kaya BS, Metin B, Tarhan N. Medication effects on EEG Biomarkers in attention-deficit/hyperactivity disorder. Clin EEG Neurosci. 2017;48:246–50. 10.1177/1550059416675232 27798290

[107] Hermens DF, Williams LM, Clarke S, Kohn M, Cooper N, Gordon E (2005). Responses to methylphenidate in adolescent AD/HD: Evidence from concurrently recorded autonomic (EDA) and central (EEG and ERP) measures. Int J Psychophysiol.

[108] Skirrow C, McLoughlin G, Banaschewski T, Brandeis D, Kuntsi J, Asherson P (2015). Normalisation of frontal theta activity following methylphenidate treatment in adult attention-deficit/hyperactivity disorder. Eur Neuropsychopharmacol.

[109] Loo SK, Bilder RM, Cho AL, Sturm A, Cowen J, Walshaw P (2016). Effects of d-methylphenidate, guanfacine, and their combination on electroencephalogram resting state spectral power in attention-deficit/hyperactivity disorder. J Am Acad Child Adolesc Psychiatry.

[110] Janssen TWP, Bink M, Geladé K, van Mourik R, Maras A, Oosterlaan J (2016). A randomized controlled trial investigating the effects of neurofeedback, methylphenidate, and physical activity on event-related potentials in children with attention-deficit/hyperactivity disorder. J Child Adolesc Psychopharmacol.

[111] Peisch V, Rutter T, Wilkinson CL, Arnett AB (2021). Sensory processing and P300 event-related potential correlates of stimulant response in children with attention-deficit/hyperactivity disorder: a critical review. Clin Neurophysiol.

[112] Rubinson M, Horowitz I, Naim-Feil J, Gothelf D, Levit-Binnun N, Moses E (2019). Effects of methylphenidate on the ERP amplitude in youth with ADHD: A double-blind placebo-controlled cross-over EEG study. PLoS ONE.

[113] Aasen IE, Øgrim G, Kropotov J, Brunner JF (2018). Methylphenidate selectively modulates one sub-component of the no-go P3 in pediatric ADHD medication responders. Biol Psychol.

[114] McGough JJ, Sturm A, Cowen J, Tung K, Salgari GC, Leuchter AF (2019). Double-blind, sham-controlled, pilot study of trigeminal nerve stimulation for attention-deficit/hyperactivity disorder. J Am Acad Child Adolesc Psychiatry.

[115] Aggensteiner P-M, Albrecht B, Strehl U, Wörz S, Ruckes C, Freitag CM (2021). Can neurophysiological markers of anticipation and attention predict ADHD severity and neurofeedback outcomes?. Biol Psychol.

[116] Aldemir R, Demirci E, Bayram AK, Canpolat M, Ozmen S, Per H (2018). EvaluatioN of two types of drug treatment with QEEG in children with ADHD. Transl Neurosci.

[117] Kratz O, Studer P, Baack J, Malcherek S, Erbe K, Moll GH (2012). Differential effects of methylphenidate and atomoxetine on attentional processes in children with ADHD: an event-related potential study using the Attention Network Test. Prog Neuropsychopharmacol Biol Psychiatry.

[118] Michelini G, Lenartowicz A, Bilder RM, McGough JJ, McCracken JT, Loo SK (2022). Methylphenidate, guanfacine, and combined treatment effects on EEG correlates of spatial working memory deficits in ADHD. J Am Acad Child Adolesc Psychiatry.

[119] Lenartowicz A, Truong H, Salgari GC, Bilder RM, McGough J, McCracken JT (2019). Alpha modulation during working memory encoding predicts neurocognitive impairment in ADHD. J Child Psychol Psychiatry.

[120] Bozhilova N, Cooper R, Kuntsi J, Asherson P, Michelini G (2020). Electrophysiological correlates of spontaneous mind wandering in attention-deficit/hyperactivity disorder. Behav Brain Res.

[121] Janssen TWP, Geladé K, Bink M, van Mourik R, Twisk JWR, Maras A (2020). Long-term effects of theta/beta neurofeedback on EEG power spectra in children with attention deficit hyperactivity disorder. Clin Neurophysiol.

[122] Cortese S (2021). Setting the foundations of developmental precision psychiatry for ADHD. Am J Psychiatry.

[123] Posner J (2018). The role of precision medicine in child psychiatry: what can we expect and when?. J Am Acad Child Adolesc Psychiatry.

[124] Loo SK, McGough JJ, McCracken JT, Smalley SL. Parsing heterogeneity in attention-deficit hyperactivity disorder using EEG-based subgroups. J Child Psychol Psychiatry. 2017. 10.1111/jcpp.12814. 10.1111/jcpp.12814 PMC5812789 28921526

[125] Button KS, Ioannidis JP, Mokrysz C, Nosek BA, Flint J, Robinson ES (2013). Power failure: why small sample size undermines the reliability of neuroscience. Nat Rev Neurosc.

[126] Marek S, Tervo-Clemmens B, Calabro FJ, Montez DF, Kay BP, Hatoum AS (2022). Reproducible brain-wide association studies require thousands of individuals. Nature.

[127] Shic F, Naples AJ, Barney EC, Chang SA, Li B, McAllister T (2022). The autism biomarkers consortium for clinical trials: evaluation of a battery of candidate eye-tracking biomarkers for use in autism clinical trials. Mol Autism.

[128] Whitton AE, Webb CA, Dillon DG, Kayser J, Rutherford A, Goer F (2019). Pretreatment rostral anterior cingulate cortex connectivity with salience network predicts depression recovery: findings from the EMBARC randomized clinical trial. Biol Psychiatry.

[129] Kittel-Schneider S, Arteaga-Henriquez G, Vasquez AA, Asherson P, Banaschewski T, Brikell I (2022). Non-mental diseases associated with ADHD across the lifespan: fidgety philipp and pippi longstocking at risk of multimorbidity?. Neurosci Biobehav Rev.

[130] Charpentier CJ, Faulkner P, Pool ER, Ly V, Tollenaar MS, Kluen LM (2021). How representative are neuroimaging samples? Large-scale evidence for trait anxiety differences between fMRI and behaviour-only research participants. Soc Cogn Affect Neurosci.

[131] Etienne A, Laroia T, Weigle H, Afelin A, Kelly SK, Krishnan A (2020). Novel electrodes for reliable EEG recordings on coarse and curly hair. Annu Int Conf IEEE Eng Med Biol Soc.

[132] Ruan Y, Lin Y-F, Feng Y-CA, Chen C-Y, Lam M, Guo Z (2022). Improving polygenic prediction in ancestrally diverse populations. Nat Genet.

[133] Goldfarb MG, Brown DR (2022). Diversifying participation: the rarity of reporting racial demographics in neuroimaging research. Neuroimage.

[134] Martin AR, Kanai M, Kamatani Y, Okada Y, Neale BM, Daly MJ (2019). Clinical use of current polygenic risk scores may exacerbate health disparities. Nat Genet.

[135] Slobodin O, Masalha R (2020). Challenges in ADHD care for ethnic minority children: a review of the current literature. Transcult Psychiatry.

[136] Bzdok D, Meyer-Lindenberg A (2018). Machine learning for precision psychiatry: opportunities and challenges. Biol Psychiatry Cogn Neurosci Neuroimaging.

[137] Kim J-W, Sharma V, Ryan ND. Predicting methylphenidate response in ADHD using machine learning approaches. International Journal of Neuropsychopharmacology. 2015;18:pyv052. 10.1093/ijnp/pyv052 PMC4756719 25964505

[138] Sibley MH, Arnold LE, Swanson JM, Hechtman LT, Kennedy TM, Owens E, et al. Variable patterns of remission from ADHD in the multimodal treatment study of ADHD. Am J Psychiatry. 2021;179:142–51. 10.1176/appi.ajp.2021.21010032 PMC8810708 34384227

[139] Coghill D, Banaschewski T, Cortese S, Asherson P, Brandeis D, Buitelaar J, et al. The management of ADHD in children and adolescents: bringing evidence to the clinic: perspective from the European ADHD Guidelines Group (EAGG). Eur Child Adolesc Psychiatry. 2021. 10.1007/s00787-021-01871-x. 10.1007/s00787-021-01871-x PMC8532460 34677682

[140] Cortese S (2019). Debate: are stimulant medications for attention-deficit/hyperactivity disorder effective in the long term?. J Am Acad Child Adolesc Psychiatry.

[141] Elliott ML, Knodt AR, Hariri AR (2021). Striving toward translation: strategies for reliable fMRI measurement. Trends Cogn Sci.

[142] Milham MP, Vogelstein J, Xu T (2021). Removing the reliability bottleneck in functional magnetic resonance imaging research to achieve clinical utility. JAMA Psychiatry.

[143] Ethridge P, Weinberg A (2018). Psychometric properties of neural responses to monetary and social rewards across development. Int J Psychophysiol.

[144] Elliott ML, Knodt AR, Ireland D, Morris ML, Poulton R, Ramrakha S (2020). What Is the test-retest reliability of common task-functional MRI measures? new empirical evidence and a meta-analysis. Psychol Sci.

[145] Nentwich M, Ai L, Madsen J, Telesford QK, Haufe S, Milham MP, et al. Functional connectivity of EEG is subject-specific, associated with phenotype, and different from fMRI. NeuroImage. 2020;218:117001. 10.1016/j.neuroimage.2020.117001 PMC7457369 32492509

[146] Cannon RL, Baldwin DR, Shaw TL, Diloreto DJ, Phillips SM, Scruggs AM (2012). Reliability of quantitative EEG (qEEG) measures and LORETA current source density at 30 days. Neurosci Lett.

[147] Rietdijk WJR, Franken IHA, Thurik AR. Internal consistency of event-related potentials associated with cognitive control: N2/P3 and ERN/Pe. PLoS ONE. 2014 10.1371/journal.pone.0102672. 10.1371/journal.pone.0102672 PMC4102542 25033272

[148] Yang Z, Telesford QK, Franco AR, Lim R, Gu S, Xu T (2021). Measurement reliability for individual differences in multilayer network dynamics: cautions and considerations. Neuroimage.

[149] Schäfer T, Schwarz MA (2019). The meaningfulness of effect sizes in psychological research: differences between sub-disciplines and the impact of potential biases. Front Psychol.

[150] Dafflon J, Da Costa PF, Váša F, Monti RP, Bzdok D, Hellyer PJ, et al. Neuroimaging: into the Multiverse. bioRxiv. 2020. 10.1101/2020.10.29.359778.

[151] Honeycutt C, Sleath B, Bush PJ, Campbell W, Tudor G (2005). Physician use of a participatory decision-making style with children with ADHD and their parents. Patient Educ Couns.

[152] Cubillo A, Smith AB, Barrett N, Giampietro V, Brammer M, Simmons A (2014). Drug-specific laterality effects on frontal lobe activation of atomoxetine and methylphenidate in attention deficit hyperactivity disorder boys during working memory. Psychol Med.

[153] Rubia K, Halari R, Mohammad A-M, Taylor E, Brammer M (2011). Methylphenidate normalizes frontocingulate underactivation during error processing in attention-deficit/hyperactivity disorder. Biol Psychiatry.

